# Supplementary material for: Follistatin‐like 1 promotes cardiac fibroblast activation and protects the heart from rupture
Source: EMBO Mol Med. 2016 May 27;8(8):949–66. doi: 10.15252/emmm.201506151 (PMC4967946; doi:10.15252/emmm.201506151)
Supplement: Supplementary file 1 — Appendix [file EMMM-8-949-s001.pdf]

EMM 2015 06151-V3
Appendix Table of Contents

Appendix figure S1. Positive correlation between tissue and serum Fstl1 and myocardial damage.

Appendix figure S2. Co-immunolocalization analysis of Fstl1 protein with multiple cell specific proteins.

Appendix figure S3. Positive correlation between cardiac tissue Fstl1 and αSMA.

Appendix figure S4. Comparable infarct scar size in the chronic phase following MI in WT and Fstl1-cfKO mice.

Appendix figure S5. Comparable fibroblast apoptosis after MI in Fstl1-cfKO and WT mice.

Appendix figure S6. Decreased capillary density in border zone of Fstl1-cfKO mice after MI.

Appendix figure S7. Comparable cardiomyocyte hypertrophy after MI in Fstl1-cfKO and WT mice.

Appendix figure S8. Attenuated upregulation of Akt and AMPK phosphorylation after MI in Fstl1-cfKO mice.

Appendix figure S1.

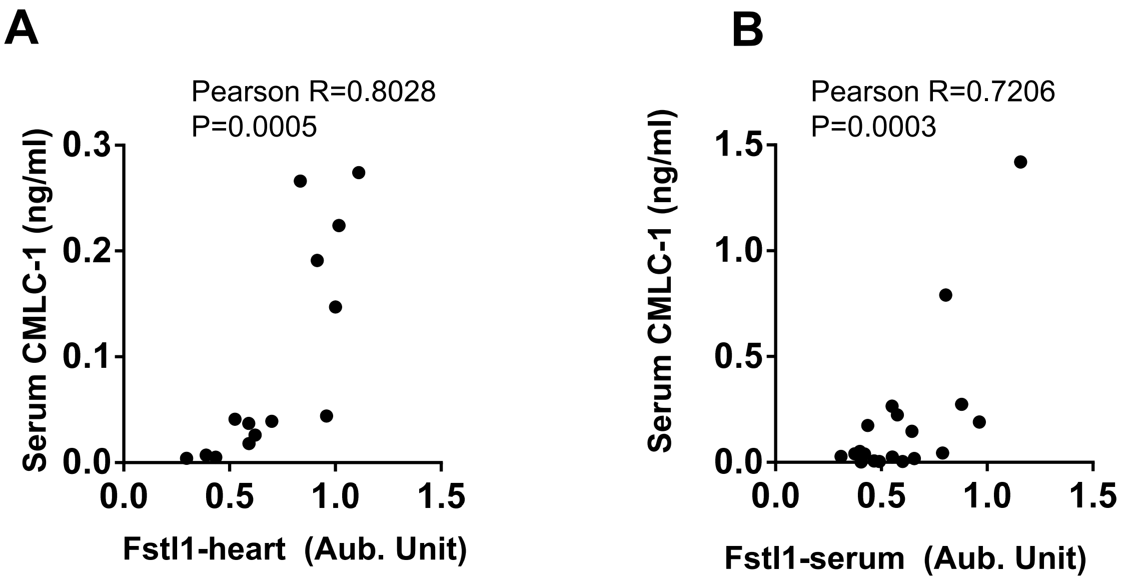

Appendix figure S1. Positive correlation between tissue and serum Fstl1 and myocardial damage. Relation between Fstl1 level in tissue (A)/ circulation (B) and cardiac injury was assessed. Heart (ischemic area) and serum Fstl1 were detected by western blotting as described in the main manuscript. Tissue Fstl1 was normalized by tubulin. Serum cardiac myosin light chain-1 (CMLC-1) was measured by ELISA kit (Life Diagnostics, Inc. Cat #CMLC-2). All samples were harvested from WT mice at day 7 after MI. Correlation was assessed by GraphPad Prism6 software (n=14 for tissue Fstl1 & CMLC-1 and n=20 for serum tissue Fstl1 & CMLC-1).

Appendix figure S2.

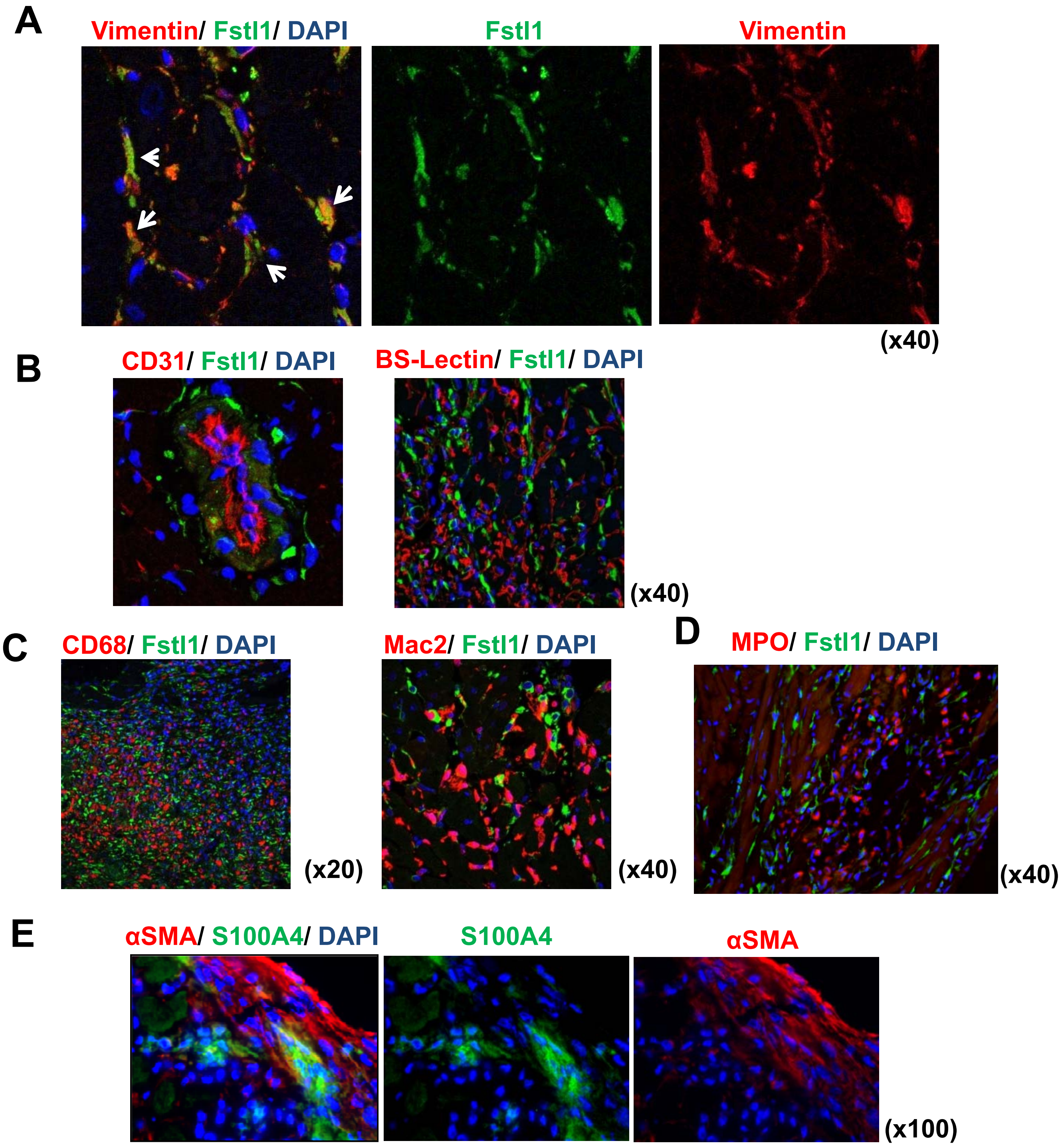

Appendix figure S2. Co-immunolocalization analysis of Fstl1 protein with multiple cell specific proteins. (A) Heart samples harvested at 7 days after MI were stained with Fstl1 and vimentin antibodies. DAPI was used for nuclei staining. Arrows indicate co-localized cells. (B) Heart samples harvested at 3 days after MI were stained with Fstl1 and CD31 (left) or BS-lectin (right) antibodies. DAPI was used for nuclei staining. (C) Heart samples harvested at 3 days after MI were stained with Fstl1 and CD68 (left) or Mac2 (right) antibodies. DAPI was used for nuclei staining. (D) Heart samples harvested a day after MI were stained with Fstl1 and MPO antibody. DAPI was used for nuclei staining. (E) Heart samples harvested at 7 days after MI were stained with S100A4 and α-SMA antibodies. DAPI was used for nuclei staining.

Appendix figure S3.

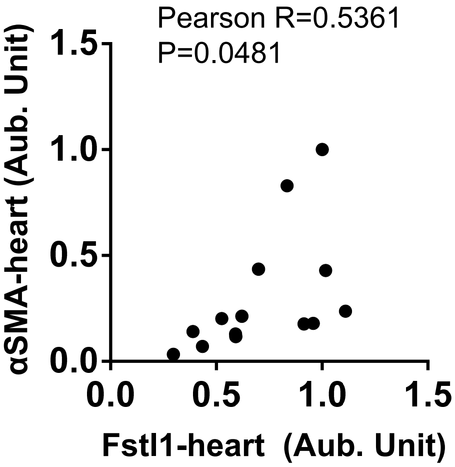

Appendix figure S3. Positive correlation between cardiac tissue Fstl1 and αSMA. The relationship between tissue Fstl1 level and αSMA expression was assessed. All samples were harvested at day 7 after MI. The ischemic myocardium of WT heart was processed as described in the main manuscript. Mouse Fstl1 and αSMA protein were detected by western blotting. Correlation was assessed by GraphPad Prism 6 software (n=14).

Appendix figure S4.

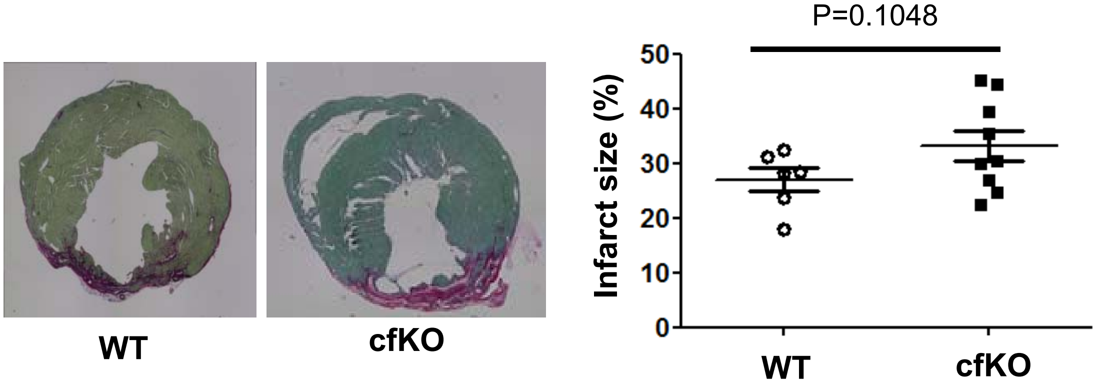

Appendix figure S4. Comparable infarct scar size in the chronic phase following MI in WT and Fstl1-cfKO mice. Heart samples were harvested at day 28 after MI and processed for paraffin histological sections. Sections were stained with Picrosirius red stain to visualize the infarcted area. The size of the infarcted area was normalized based upon the whole heart size in the section. Error bars represent mean ± SEM (n= 6 and 9 for WT and cfKO, respectively). Statistical analysis was performed by unpaired t-test (two-tailed).

Appendix figure S5.

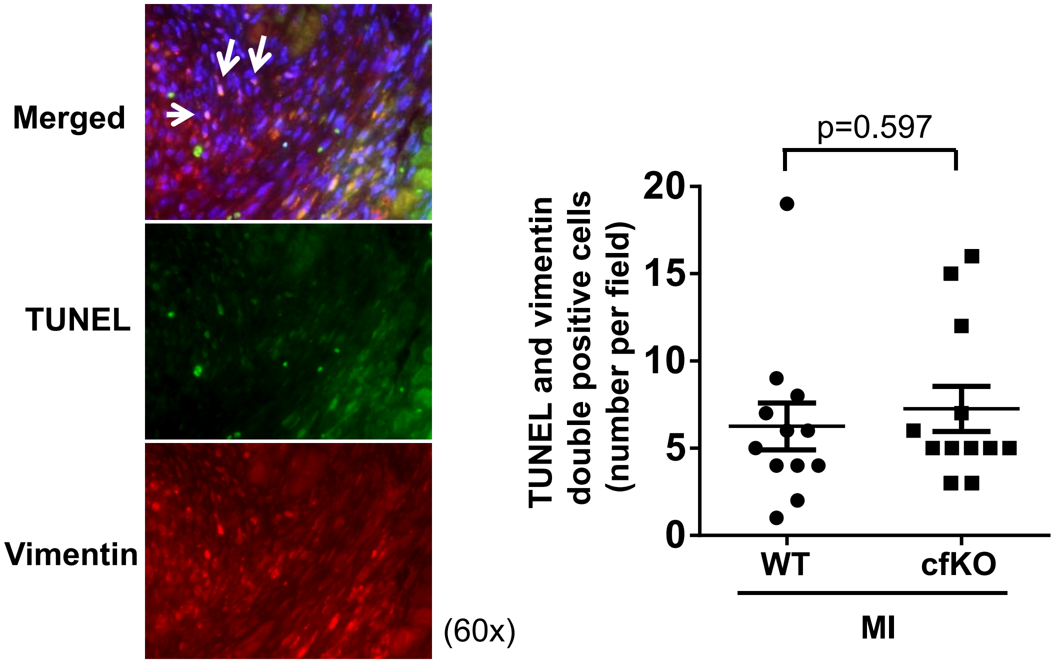

Appendix figure S5. Comparable fibroblast apoptosis after MI in Fstl1-cfKO and WT mice. Fibroblast apoptosis was assessed by TUNEL and vimentin co-staining. WT and cfKO hearts sampled at day 7 after MI surgery were used. DAPI was used for nuclei staining. TUNEL staining was performed using FITC-conjugated *in Situ* Cell Death Detection Kit (Roche) following the manufacturer's protocol. Vimentin was detected using anti-vimentin antibody (Santa Cruz Biotechnology, H-84) and secondary donkey anti-rabbit AF598 conjugated antibody (Life Technologies). DAPI was used for nuclei staining. TUNEL and vimentin double positive cells in the infarcted area and border zone were counted at high magnification. Error bars represent mean ± SEM (n=12 for WT and cfKO).Statistical analysis was performed by unpaired t-test (two-tailed).

Appendix figure S6.

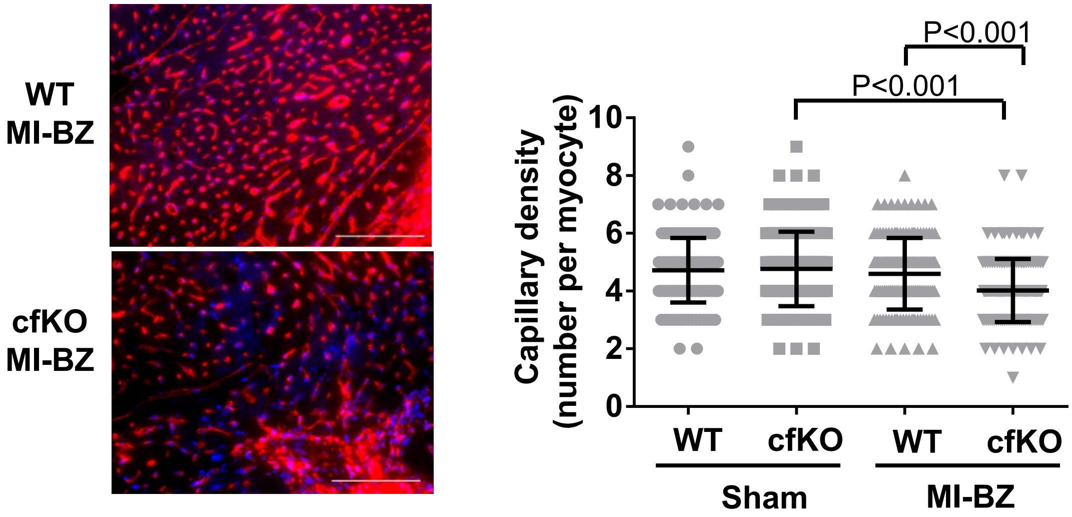

Appendix figure S6. Decreased capillary density in border zone of Fstl1-cfKO mice after MI. Capillary density was determined by staining with Alexa-594 conjugated Isolectin-IB4 antibody (Life Technologies) in WT and cfKO heart at day 7 after sham or MI surgery. DAPI was used for nuclei staining. Capillary number per cardiomyocyte was measured in the border zone of infarcted heart and the left ventricle of sham heart. Error bars represent mean ± SEM (n=150 cardiomyocytes for each group).Statistical analysis was performed by Kruskal-Wallis test and Dunn's test for post hoc analysis.

Appendix figure S7.

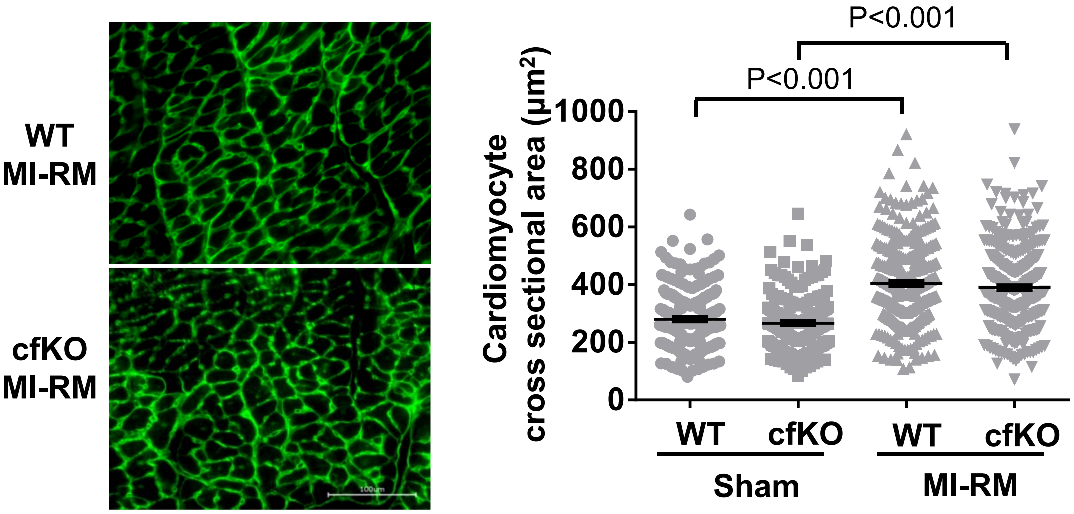

Appendix figure S7. Comparable cardiomyocyte hypertrophy after MI in Fstl1-cfKO and WT mice. Cardiomyocyte hypertrophy was assessed by measuring cardiomyocyte cross-sectional area of non-ischemic myocardium at 7 days after MI. Paraffin sections were stained using FITC-conjugated GWA antibody (Life Technologies) to detect the outline of the cardiomyocytes. Error bars represent mean ± SEM (n=150 cardiomyocytes for each group). Statistical analysis was performed by Kruskal-Wallis and Dunn's test for post hoc analysis.

Appendix figure S8.

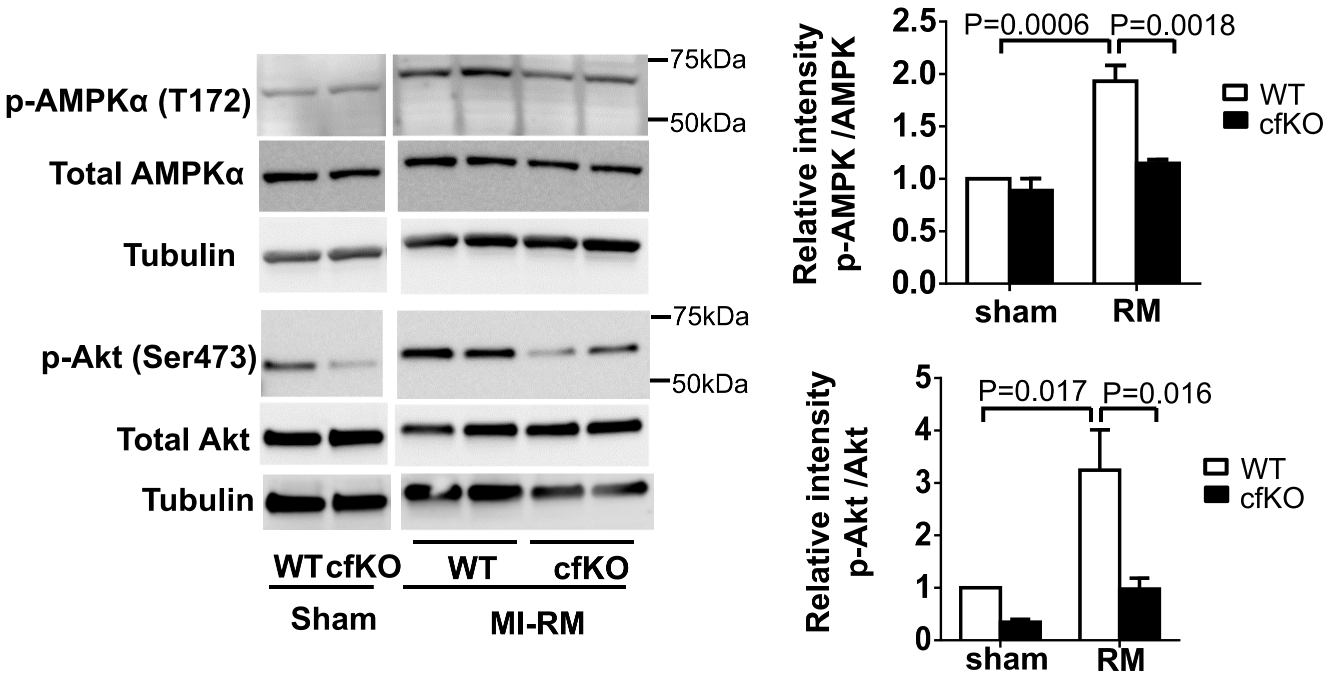

Appendix figure S8. Attenuated upregulation of Akt and AMPK phosphorylation after MI in Fstl1-cfKO mice. Detection of AMPK and Akt signals in the heart at day 7 after sham or MI surgery. All antibodies were purchased from Cell Signaling: p-AMPKα (T172) antibody (Cat. #2535), AMPKα antibody (Cat. #2532), p-Akt (Ser473) antibody (Cat. #4058), Akt antibody (Cat. #9272) and tubulin antibody (Cat. #2148). Error bars represent mean ± SEM (n=3 for each group). Statistical analysis was performed by two-way ANOVA and Tukey's test for post hoc analysis.
