# Supplementary material for: Follistatin‐like 1 promotes cardiac fibroblast activation and protects the heart from rupture
Source: EMBO Mol Med. 2016 May 27;8(8):949–66. doi: 10.15252/emmm.201506151 (PMC4967946; doi:10.15252/emmm.201506151)
Supplement: Supplementary file 2 — Expanded View Figures PDF [file EMMM-8-949-s002.pdf]

Expanded View Figures

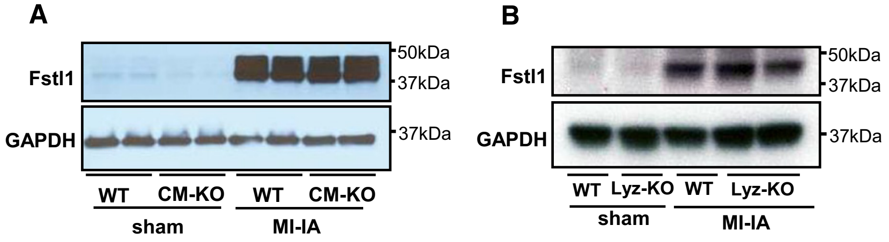

Figure EV1. Neither cardiomyocytes nor myeloid cells are responsible for the induction of Fstl1 in the post-MI heart.

A MI-induced Fstl1 protein induction in cardiomyocyte-specific Fstl1 knockout mice (αMHC$^{cre+/-}$ × Fstl1$^{flox/flox}$) and littermate WT mice (αMHC$^{cre-/-}$ × Fstl1$^{flox/flox}$) was assessed. The heart was sampled at 7 days after MI. No attenuation of MI-induced Fstl1 expression was observed.

B MI-induced Fstl1 protein expression in myeloid cell-specific Fstl1 knockout mice (LyzM$^{cre+/-}$ × Fstl1$^{flox/flox}$) and littermate WT mice (LyzM$^{cre-/-}$ × Fstl1$^{flox/flox}$) was assessed. The heart was sampled at 7 days after MI. MI-induced cardiac Fstl1 expression was not attenuated.

Source data are available online for this figure.

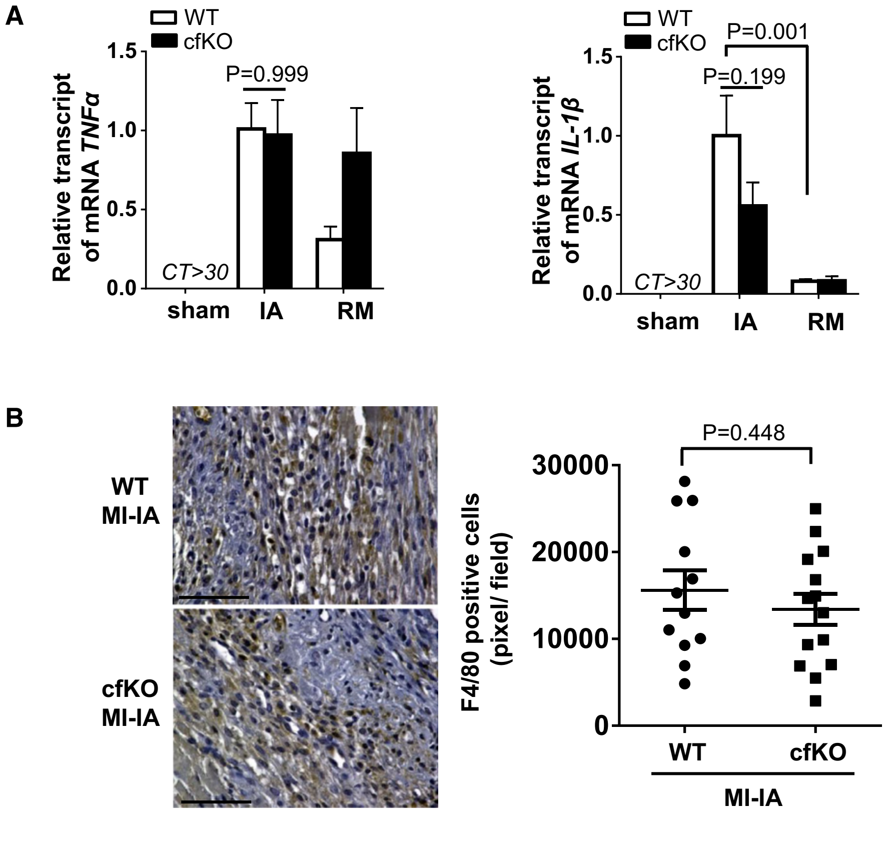

Figure EV2. Comparable inflammatory response after MI in Fstl1-cfKO and WT mice.

A qPCR analysis of TNF-α and IL-1β mRNA expression in heart tissues. The heart was harvested at 7 days after the surgery. Error bars represent mean ± SEM (n = 16 and 15 for WT and cfKO sham group, n = 15 and 14 for WT and cfKO MI group, respectively). Statistical analysis was performed by two-way ANOVA. *Post hoc* test was performed by Tukey's test.

B Detection of macrophage by F4/80 staining (AbD Serotec, Clone A3-1) in the ischemic myocardium of WT and cfKO mice at day 7 after MI. DAB substrate was used for detection. Counter staining was performed using hematoxylin stain. F4/80-positive cell density in the infarcted area was measured as pixel at high magnification. Scale bar indicates 100 μm. Error bars represent mean ± SEM (n = 12 and 14 for WT and cfKO, respectively). Statistical analysis was performed by unpaired t-test (two-tailed).

Source data are available online for this figure.

© 2016 The Authors

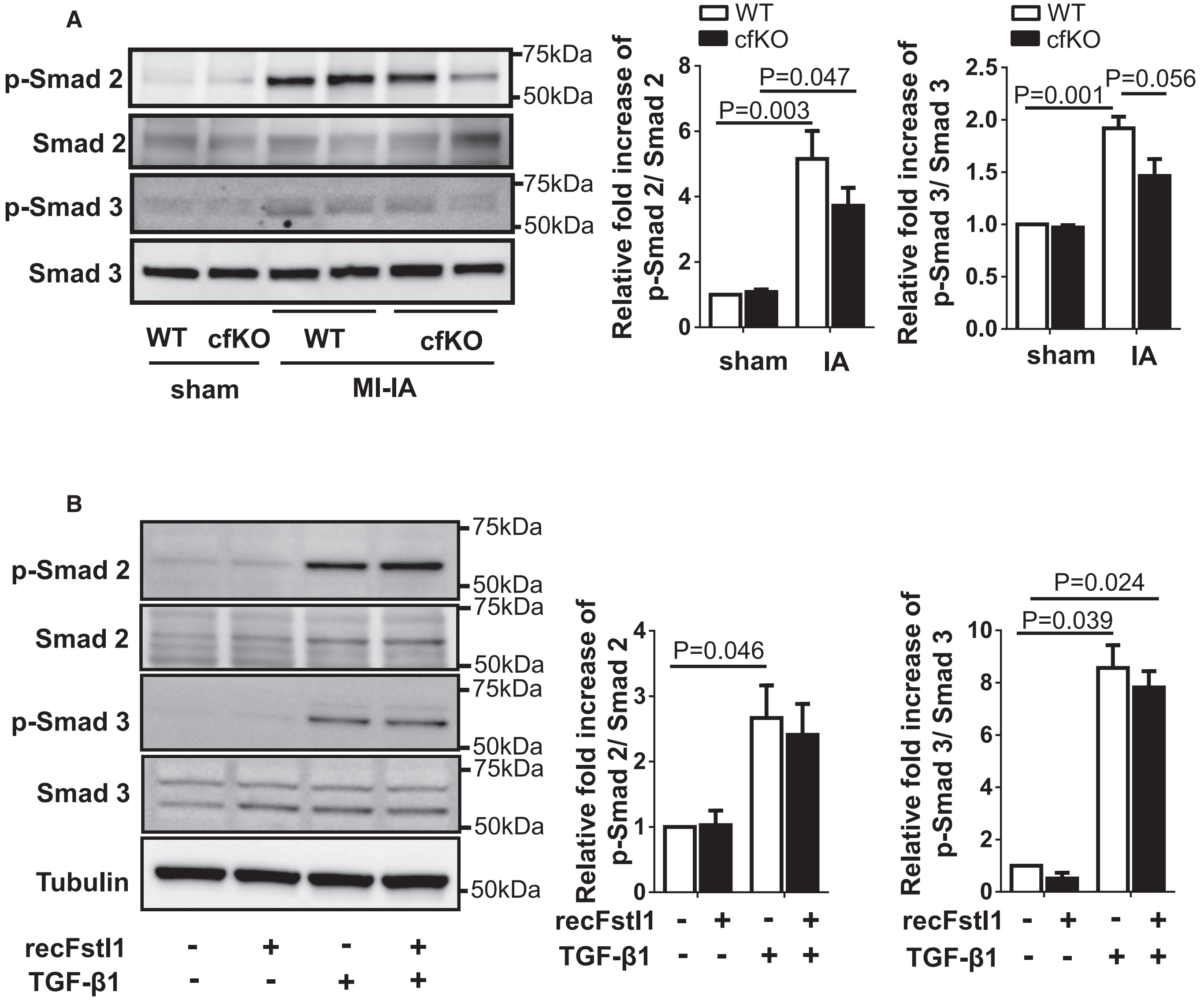

Figure EV3. Impact of Fstl1 on Smad2/3 signaling in infarcted hearts and cultured fibroblasts.

A The expression of p-Smad2, p-Smad3, Smad2, and Smad3 in Fstl1-cfKO and WT hearts was assessed by immunoblotting. Hearts were harvested at 7 days after the surgery. Error bars represent mean ± SEM (n = 3 for each sham group and n = 4 for each MI-IA group). Statistical analysis was performed by two-way ANOVA. *Post hoc* test was performed by Tukey's test.

B The effect of recombinant Fstl1 protein on TGF-β1-stimulated Smad2/3 signaling pathway in neonatal cardiac fibroblasts (NRCFbs). Recombinant Fstl1 protein (50 ng/ml) or control vehicle was added to serum-deprived (24 h) NRCFbs at 30 min prior to TGF-β1 protein (2 ng/ml) stimulation. Samples were harvested at 15 min after TGF-β1 stimulation. Phosphorylation of Smad 2/3 and Smad 2/3 expression in cell lysates was assessed by immunoblotting. Tubulin was used for internal control. Error bars represent mean ± SEM (n = 3 for each group). Statistical analysis was performed by ordinary one-way ANOVA and Tukey's test for Smad 2, Kruskal–Wallis test and Dunnett's T3 test for Smad 3. Three independent experiments were performed.

Source data are available online for this figure.

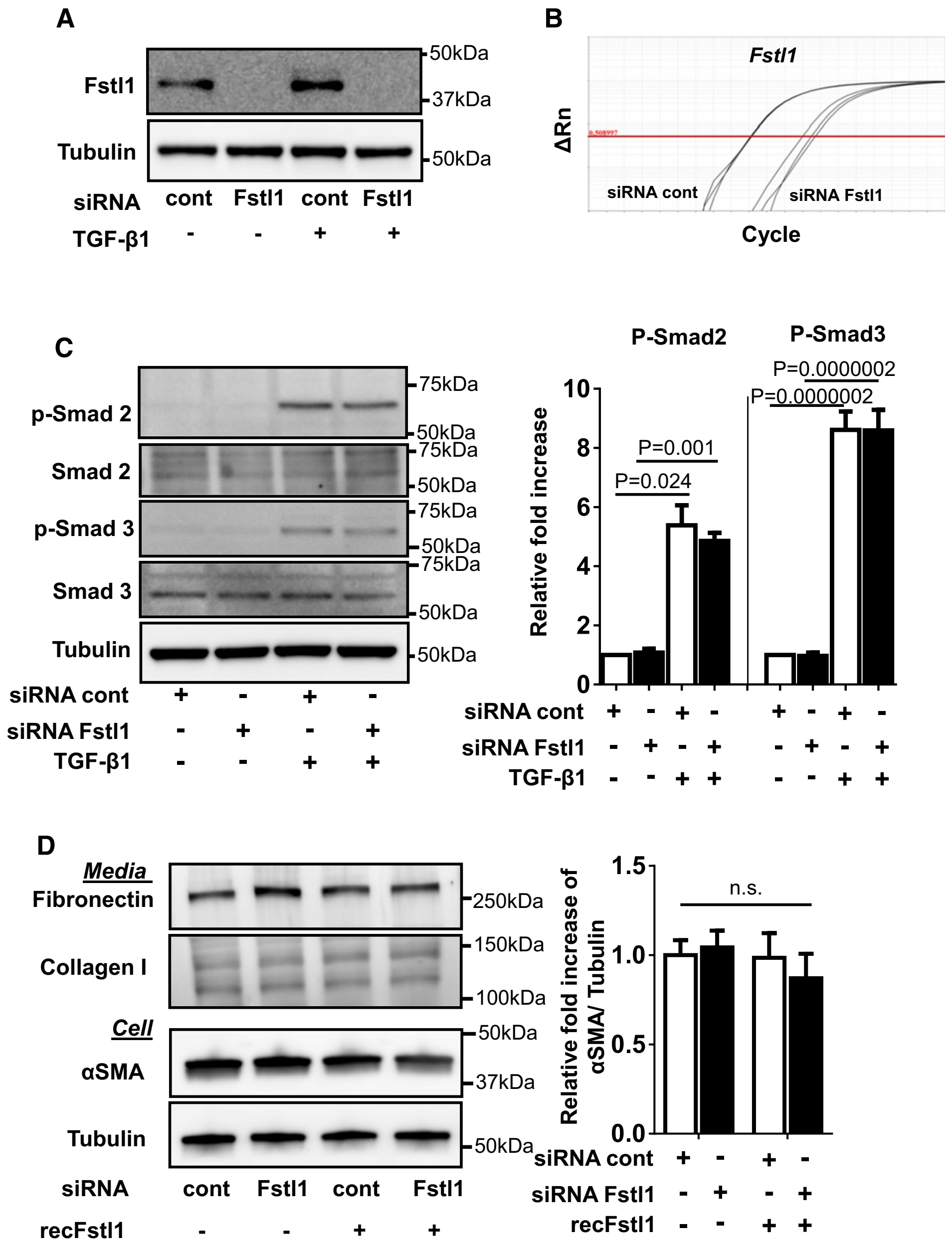

Figure EV4. Endogenous Fstl1 does not contribute to TGF-β 1/Smad 2/3 signaling or myofibroblast transdifferentiation.

A Fstl1 ablation by siRNA Fstl1 (6 pmol) in NRCFbs. siRNA Fstl1 or siRNA non-targeting negative control was transfected to NRCFbs by lipofectamine RNAimax for 12 h, followed by stimulation of recombinant TGF-β1 (10 ng/ml) for 24 h. Fstl1 protein expression in cell lysate was analyzed by immunoblotting.

B Efficiency of Fstl1 gene ablation by siRNA Fstl1 (6 pmol) in NRCFbs. siRNA Fstl1 or siRNA non-targeting negative control was transfected to NRCFbs by lipofectamine RNAimax for 42 h. mRNA transcripts of Fstl1 and GAPDH were assessed by qPCR. The amplification plots for Fstl1 are shown. The Ct values for Fstl1 in siRNA control and siRNA Fstl1 are 18.17 ± 0.053 and 24.76 ± 0.420, respectively (*n* = 3 for each sample group).

C Effect of endogenous Fstl1 protein on TGF-β1-induced Smad2/3 signaling pathway in NRCFbs. Endogenous Fstl1 protein was ablated by transfecting siRNA Fstl1 to NRCFbs. siRNA negative control was used for control. Following FBS starvation for 24 h, cells were stimulated with recombinant TGF-β1 protein (2 ng/ml) for 15 min. Phosphorylation of Smad2 and Smad3 was assessed by immunoblotting. Error bars represent mean ± SEM (*n* = 3 for each group). Statistical analysis was performed by ordinary one-way ANOVA. *Post hoc* test was performed by Dunnett's T3 test for Smad2 and Tukey's test for Smad3. Two independent experiments were performed.

D Ablation of endogenous Fstl1 does not affect markers of myofibroblast differentiation or ECM protein synthesis. Endogenous Fstl1 was ablated by siRNA, and following FBS depletion for 24 h, cells were stimulated with recombinant Fstl1 protein (50 ng/ml) for 24 h. α-SMA protein expression in cell lysate and fibronectin and collagen I protein in the cultured media were assessed by immunoblotting. Tubulin was used as an internal control. Error bars represent mean ± SEM (*n* = 4 for each group). Statistical analysis was performed by ordinary one-way ANOVA and Tukey's test. Two independent experiments were performed.

Source data are available online for this figure.

 © 2016 The Authors

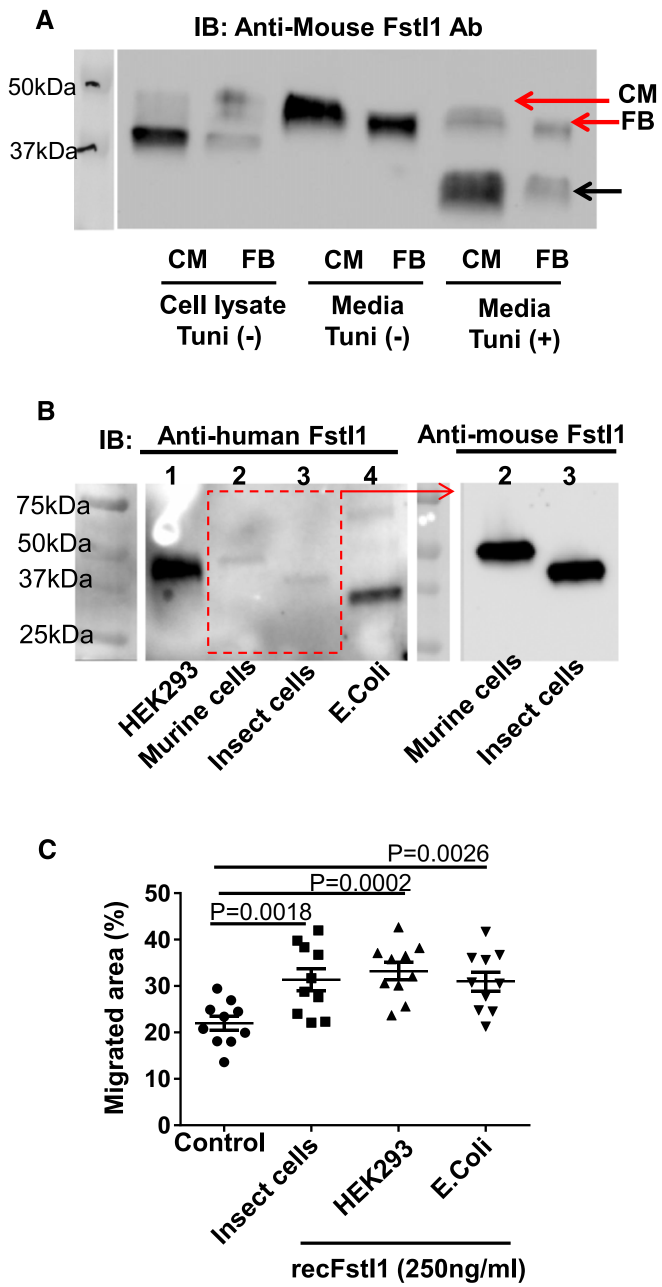

Figure EV5. Differential Fstl1 glycosylation and its impact on bioactivity.

A Western blot analysis of mouse Fstl1 protein in cell lysates and secreted from cardiomyocytes and cardiac fibroblasts. Neonatal rat cardiomyocytes and cardiac fibroblasts were infected with adenovirus encoding mouse Fstl1 (50 MOI) for 24 h. Culture media was changed from FBS 10% contained DMEM/F-12 to 0% FBS for CM and 0.5% FBS for FB. Cells were cultured with or without tunicamycin (1 μg/ml) for 16 h. Conditioned media was concentrated by Amicon Ultra filter 10k device (14,000× *g*, 10 min). Mouse Fstl1 protein was detected by Western blotting.

B Molecular size of multiple Fstl1 recombinant proteins was assessed by Western blotting. Detailed information for each recombinant protein is listed in Table EV5. Equal amount of proteins (5 ng/lane) were loaded to 4–12% TGX gel and transferred to PVDF membrane. Fstl1 proteins were detected using human and mouse Fstl1 polyclonal antibodies (both from R&D Systems).

C Bioactivity of Fstl1 recombinant proteins from different cell sources was assessed using a cardiac fibroblast migration assay. This scratch assay was performed as described in the main manuscript. Error bars represent mean ± SEM (n = 10 for each group). Statistical analysis was performed by ordinary one-way ANOVA and Fisher's LSD test for *post hoc* analysis.

Source data are available online for this figure.

© 2016 The Authors
