# Supplementary material for: Follistatin‐like 1 promotes cardiac fibroblast activation and protects the heart from rupture
Source: EMBO Mol Med. 2016 May 27;8(8):949–66. doi: 10.15252/emmm.201506151 (PMC4967946; doi:10.15252/emmm.201506151)
Supplement: Supplementary file 3 — Table EV1 [file EMMM-8-949-s003.pptx]

## Slide 1
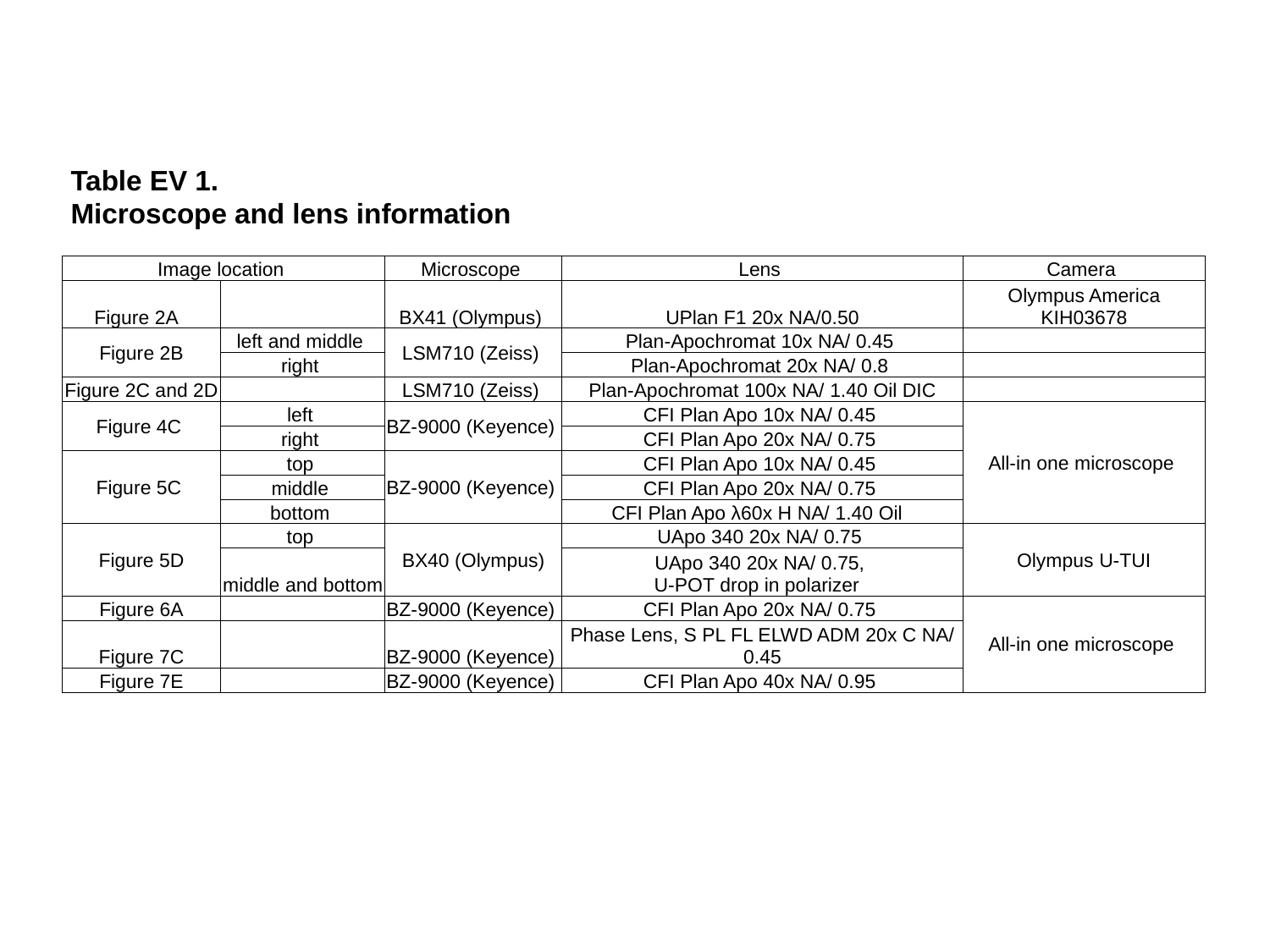

Table EV 1.
Microscope and lens information
| Image location | | Microscope | Lens | Camera |
| --- | --- | --- | --- | --- |
| Figure 2A | | BX41 (Olympus) | UPlan F1 20x NA/0.50 | Olympus America KIH03678 |
| Figure 2B | left and middle | LSM710 (Zeiss) | Plan-Apochromat 10x NA/ 0.45 | |
| | right | | Plan-Apochromat 20x NA/ 0.8 | |
| Figure 2C and 2D | | LSM710 (Zeiss) | Plan-Apochromat 100x NA/ 1.40 Oil DIC | |
| Figure 4C | left | BZ-9000 (Keyence) | CFI Plan Apo 10x NA/ 0.45 | All-in one microscope |
| | right | | CFI Plan Apo 20x NA/ 0.75 | |
| Figure 5C | top | BZ-9000 (Keyence) | CFI Plan Apo 10x NA/ 0.45 | |
| | middle | | CFI Plan Apo 20x NA/ 0.75 | |
| | bottom | | CFI Plan Apo λ60x H NA/ 1.40 Oil | |
| Figure 5D | top | BX40 (Olympus) | UApo 340 20x NA/ 0.75 | Olympus U-TUI |
| | middle and bottom | | UApo 340 20x NA/ 0.75, U-POT drop in polarizer | |
| Figure 6A | | BZ-9000 (Keyence) | CFI Plan Apo 20x NA/ 0.75 | All-in one microscope |
| Figure 7C | | BZ-9000 (Keyence) | Phase Lens, S PL FL ELWD ADM 20x C NA/ 0.45 | |
| Figure 7E | | BZ-9000 (Keyence) | CFI Plan Apo 40x NA/ 0.95 | |
