# Supplementary material for: Follistatin‐like 1 promotes cardiac fibroblast activation and protects the heart from rupture
Source: EMBO Mol Med. 2016 May 27;8(8):949–66. doi: 10.15252/emmm.201506151 (PMC4967946; doi:10.15252/emmm.201506151)
Supplement: Supplementary file 4 — Table EV2 [file EMMM-8-949-s004.pptx]

## Slide 1
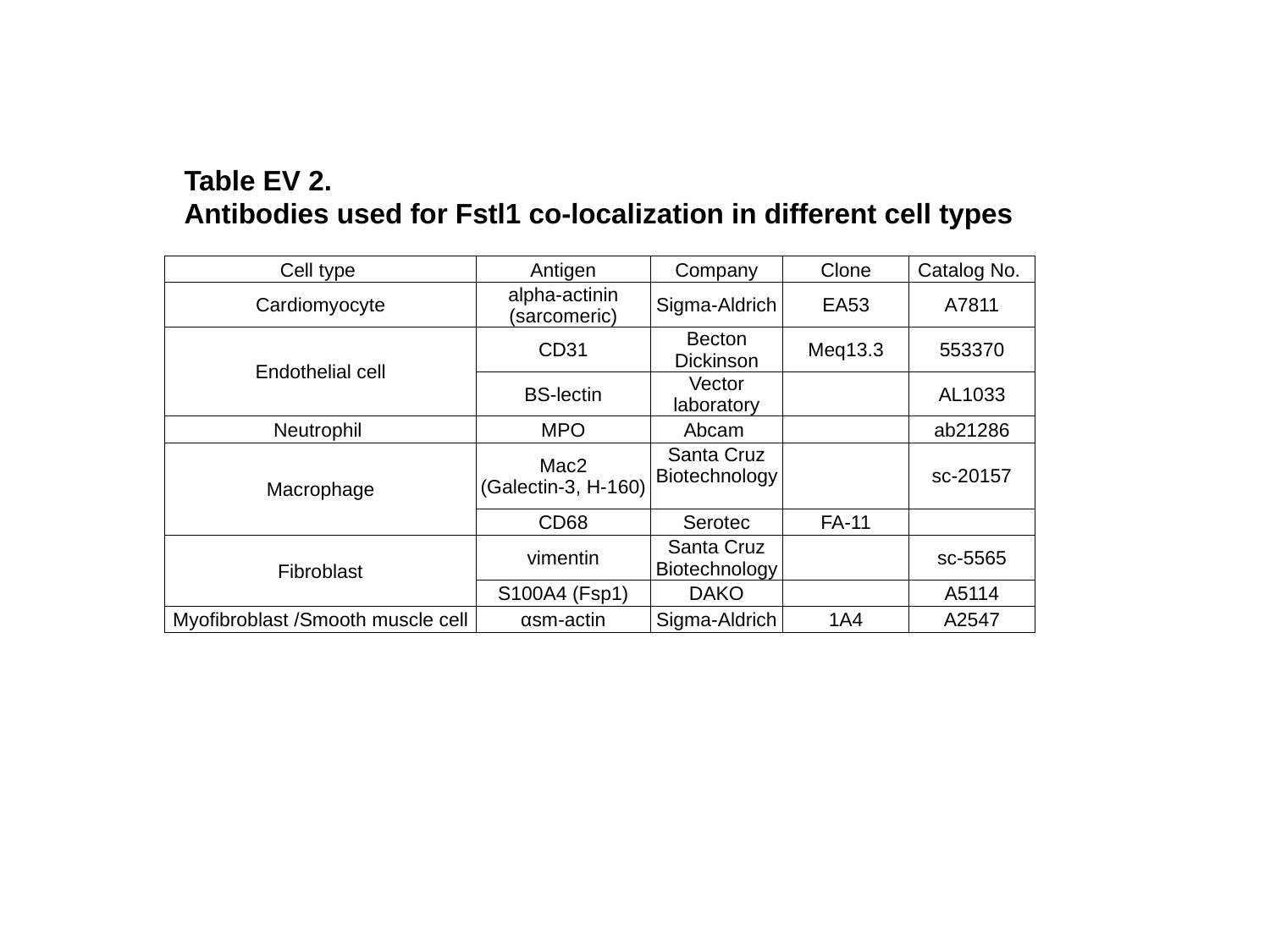

Table EV 2.
Antibodies used for Fstl1 co-localization in different cell types
| Cell type | Antigen | Company | Clone | Catalog No. |
| --- | --- | --- | --- | --- |
| Cardiomyocyte | alpha-actinin (sarcomeric) | Sigma-Aldrich | EA53 | A7811 |
| Endothelial cell | CD31 | Becton Dickinson | Meq13.3 | 553370 |
| | BS-lectin | Vector laboratory | | AL1033 |
| Neutrophil | MPO | Abcam | | ab21286 |
| Macrophage | Mac2 (Galectin-3, H-160) | Santa Cruz Biotechnology | | sc-20157 |
| | CD68 | Serotec | FA-11 | |
| Fibroblast | vimentin | Santa Cruz Biotechnology | | sc-5565 |
| | S100A4 (Fsp1) | DAKO | | A5114 |
| Myofibroblast /Smooth muscle cell | αsm-actin | Sigma-Aldrich | 1A4 | A2547 |
