# Supplementary material for: Follistatin‐like 1 promotes cardiac fibroblast activation and protects the heart from rupture
Source: EMBO Mol Med. 2016 May 27;8(8):949–66. doi: 10.15252/emmm.201506151 (PMC4967946; doi:10.15252/emmm.201506151)
Supplement: Supplementary file 5 — Table EV3 [file EMMM-8-949-s005.pptx]

## Slide 1
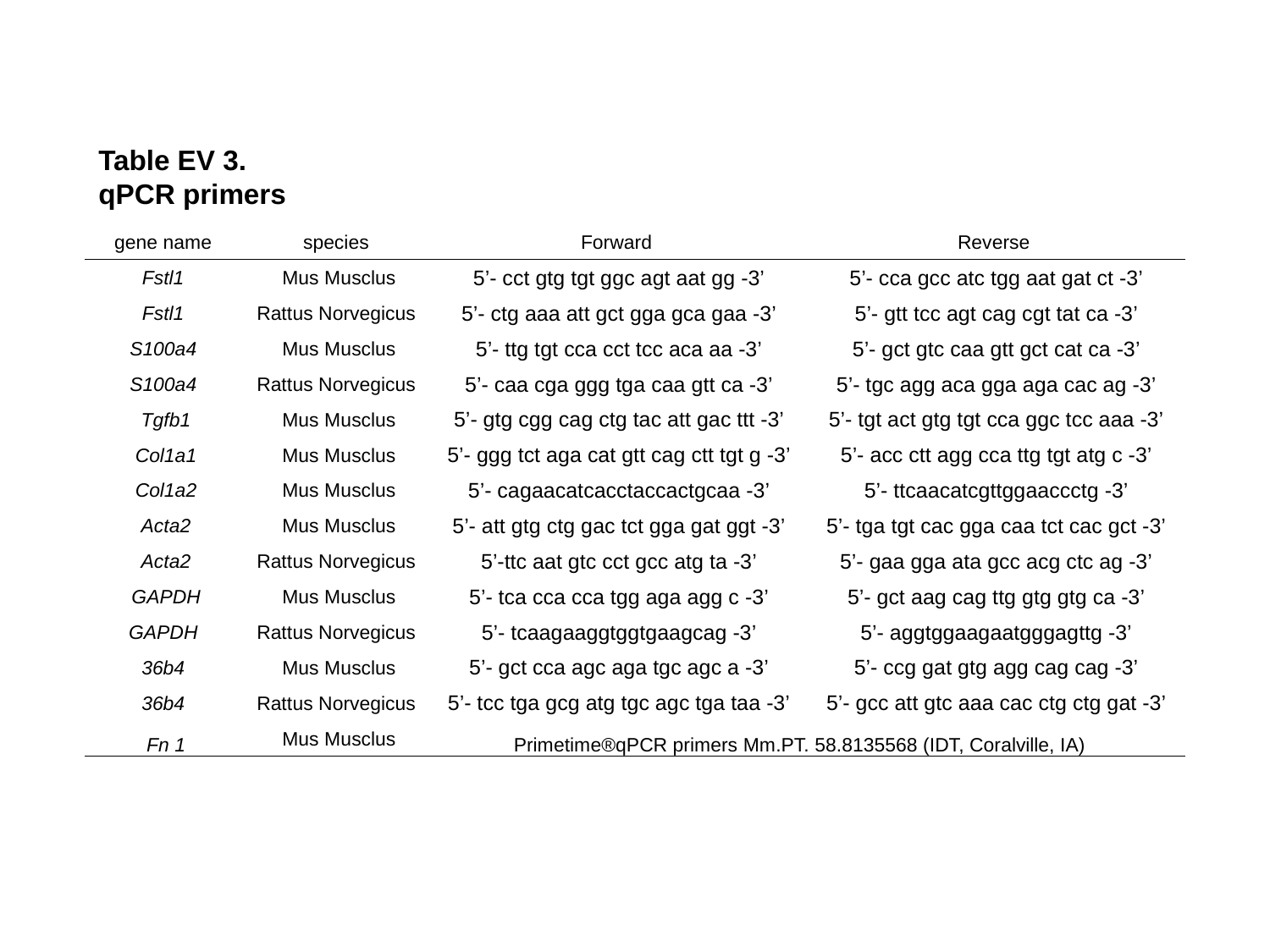

Table EV 3.
qPCR primers
| gene name | species | Forward | Reverse |
| --- | --- | --- | --- |
| Fstl1 | Mus Musclus | 5’- cct gtg tgt ggc agt aat gg -3’ | 5’- cca gcc atc tgg aat gat ct -3’ |
| Fstl1 | Rattus Norvegicus | 5’- ctg aaa att gct gga gca gaa -3’ | 5’- gtt tcc agt cag cgt tat ca -3’ |
| S100a4 | Mus Musclus | 5’- ttg tgt cca cct tcc aca aa -3’ | 5’- gct gtc caa gtt gct cat ca -3’ |
| S100a4 | Rattus Norvegicus | 5’- caa cga ggg tga caa gtt ca -3’ | 5’- tgc agg aca gga aga cac ag -3’ |
| Tgfb1 | Mus Musclus | 5’- gtg cgg cag ctg tac att gac ttt -3’ | 5’- tgt act gtg tgt cca ggc tcc aaa -3’ |
| Col1a1 | Mus Musclus | 5’- ggg tct aga cat gtt cag ctt tgt g -3’ | 5’- acc ctt agg cca ttg tgt atg c -3’ |
| Col1a2 | Mus Musclus | 5’- cagaacatcacctaccactgcaa -3’ | 5’- ttcaacatcgttggaaccctg -3’ |
| Acta2 | Mus Musclus | 5’- att gtg ctg gac tct gga gat ggt -3’ | 5’- tga tgt cac gga caa tct cac gct -3’ |
| Acta2 | Rattus Norvegicus | 5’-ttc aat gtc cct gcc atg ta -3’ | 5’- gaa gga ata gcc acg ctc ag -3’ |
| GAPDH | Mus Musclus | 5’- tca cca cca tgg aga agg c -3’ | 5’- gct aag cag ttg gtg gtg ca -3’ |
| GAPDH | Rattus Norvegicus | 5’- tcaagaaggtggtgaagcag -3’ | 5’- aggtggaagaatgggagttg -3’ |
| 36b4 | Mus Musclus | 5’- gct cca agc aga tgc agc a -3’ | 5’- ccg gat gtg agg cag cag -3’ |
| 36b4 | Rattus Norvegicus | 5’- tcc tga gcg atg tgc agc tga taa -3’ | 5’- gcc att gtc aaa cac ctg ctg gat -3’ |
| Fn 1 | Mus Musclus | Primetime®qPCR primers Mm.PT. 58.8135568 (IDT, Coralville, IA) | |
