# Supplementary material for: Follistatin‐like 1 promotes cardiac fibroblast activation and protects the heart from rupture
Source: EMBO Mol Med. 2016 May 27;8(8):949–66. doi: 10.15252/emmm.201506151 (PMC4967946; doi:10.15252/emmm.201506151)
Supplement: Supplementary file 6 — Table EV4 [file EMMM-8-949-s006.pptx]

## Slide 1
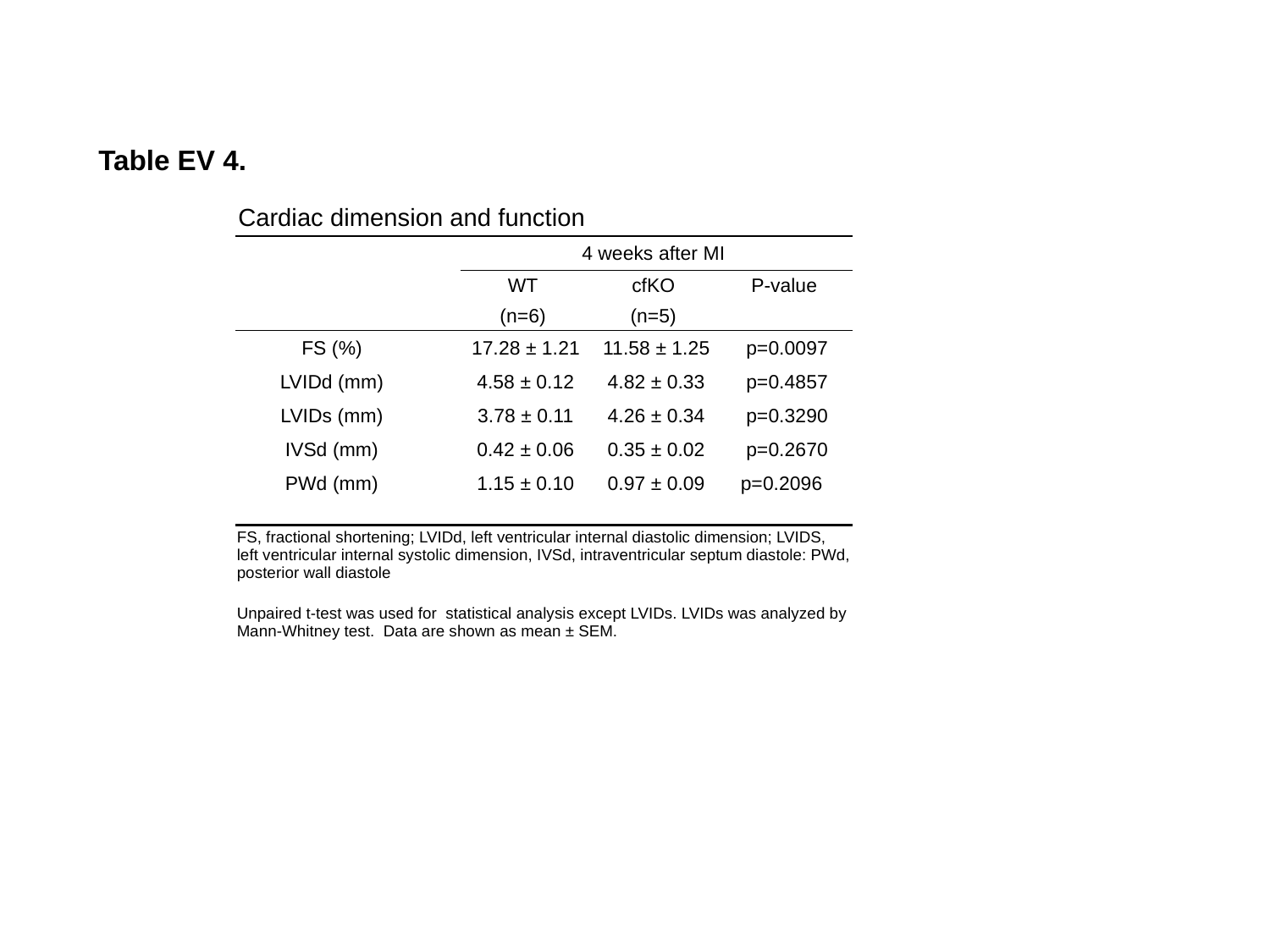

Table EV 4.
Cardiac dimension and function
| | | | | |
| --- | --- | --- | --- | --- |
| | | 4 weeks after MI | | |
| | | WT | cfKO | P-value |
| | | (n=6) | (n=5) | |
| FS (%) | | 17.28 ± 1.21 | 11.58 ± 1.25 | p=0.0097 |
| LVIDd (mm) | | 4.58 ± 0.12 | 4.82 ± 0.33 | p=0.4857 |
| LVIDs (mm) | | 3.78 ± 0.11 | 4.26 ± 0.34 | p=0.3290 |
| IVSd (mm) | | 0.42 ± 0.06 | 0.35 ± 0.02 | p=0.2670 |
| PWd (mm) | | 1.15 ± 0.10 | 0.97 ± 0.09 | p=0.2096 |
| | | | | |
| FS, fractional shortening; LVIDd, left ventricular internal diastolic dimension; LVIDS, left ventricular internal systolic dimension, IVSd, intraventricular septum diastole: PWd, posterior wall diastole | | | | |
| Unpaired t-test was used for statistical analysis except LVIDs. LVIDs was analyzed by Mann-Whitney test. Data are shown as mean ± SEM. | | | | |
| | | | | |
