# Supplementary material for: Follistatin‐like 1 promotes cardiac fibroblast activation and protects the heart from rupture
Source: EMBO Mol Med. 2016 May 27;8(8):949–66. doi: 10.15252/emmm.201506151 (PMC4967946; doi:10.15252/emmm.201506151)
Supplement: Supplementary file 7 — Table EV5 [file EMMM-8-949-s007.pptx]

## Slide 1
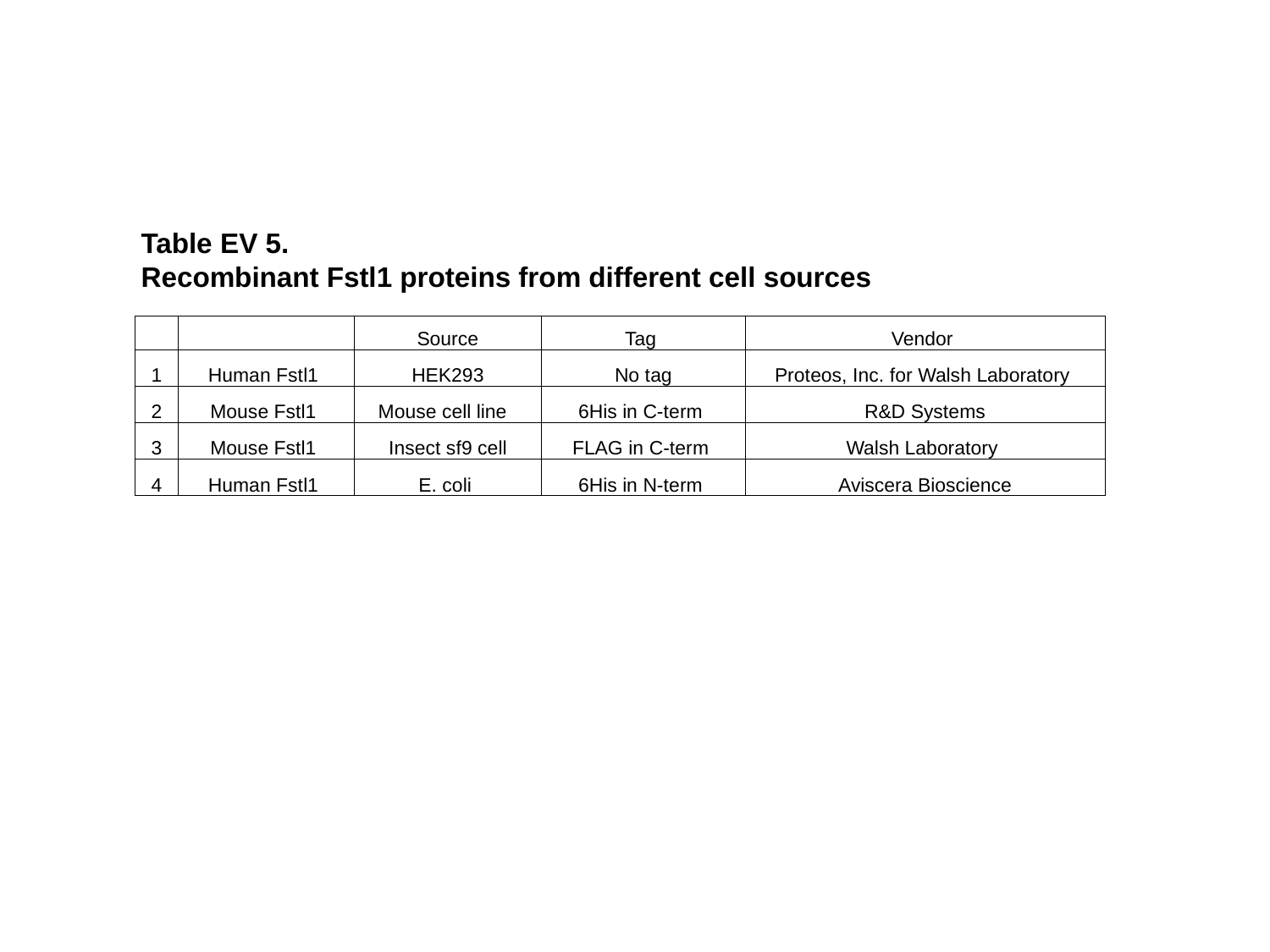

Table EV 5.
Recombinant Fstl1 proteins from different cell sources
| | | Source | Tag | Vendor |
| --- | --- | --- | --- | --- |
| 1 | Human Fstl1 | HEK293 | No tag | Proteos, Inc. for Walsh Laboratory |
| 2 | Mouse Fstl1 | Mouse cell line | 6His in C-term | R&D Systems |
| 3 | Mouse Fstl1 | Insect sf9 cell | FLAG in C-term | Walsh Laboratory |
| 4 | Human Fstl1 | E. coli | 6His in N-term | Aviscera Bioscience |
