# Supplementary material for: Follistatin‐like 1 promotes cardiac fibroblast activation and protects the heart from rupture
Source: EMBO Mol Med. 2016 May 27;8(8):949–66. doi: 10.15252/emmm.201506151 (PMC4967946; doi:10.15252/emmm.201506151)
Supplement: Supplementary file 8 — Source Data for Expanded View and Appendix [file EMMM-8-949-s008.zip › Source_Data_For_EV_And_Appendix/Appendix_Figure_S8_Source_data.pptx]

## Slide 1
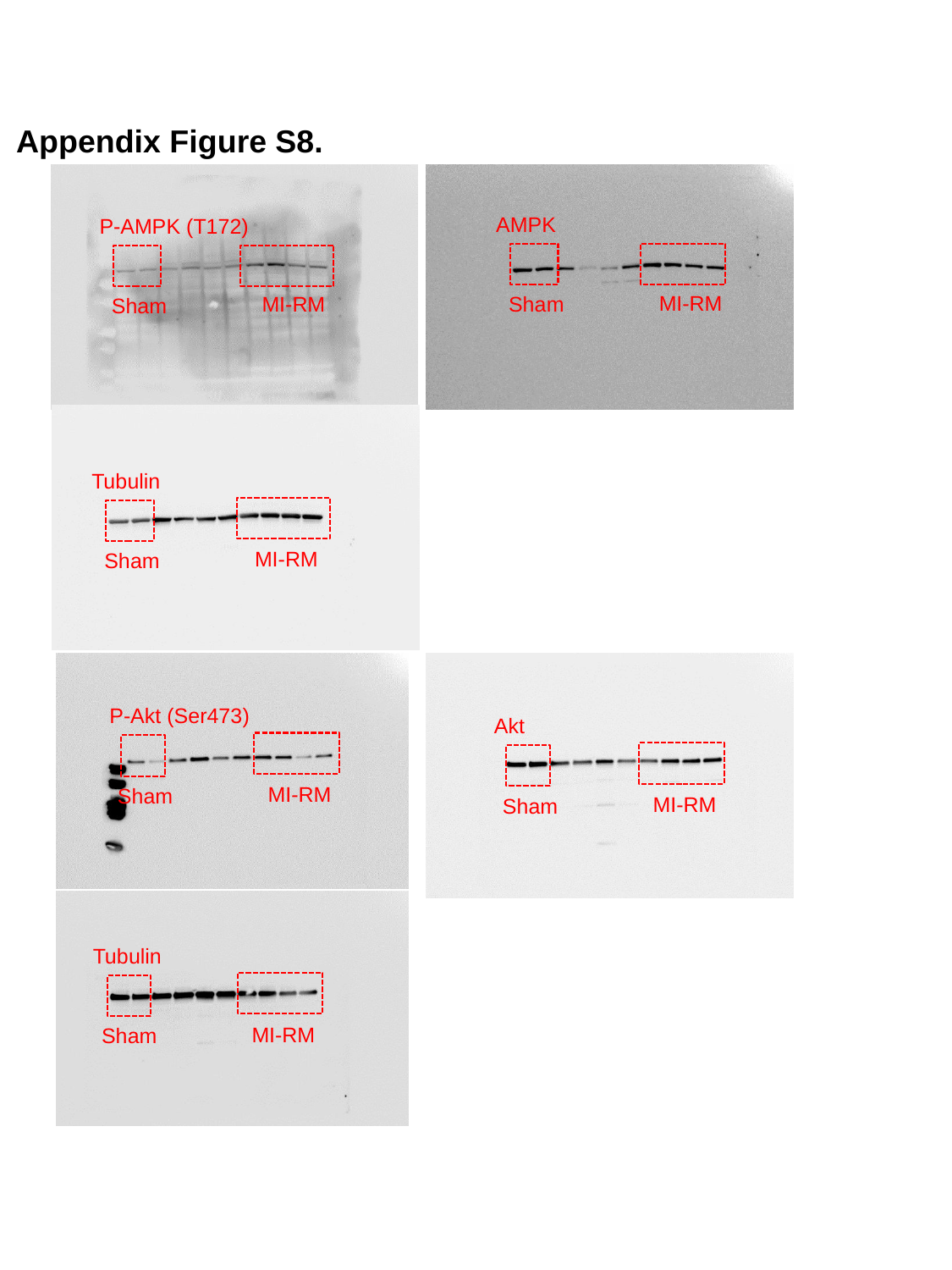

Appendix Figure S8.
AMPK
P-AMPK (T172)
MI-RM
MI-RM
Sham
Sham
Tubulin
MI-RM
Sham
P-Akt (Ser473)
Akt
MI-RM
Sham
MI-RM
Sham
Tubulin
MI-RM
Sham

## Slide 2
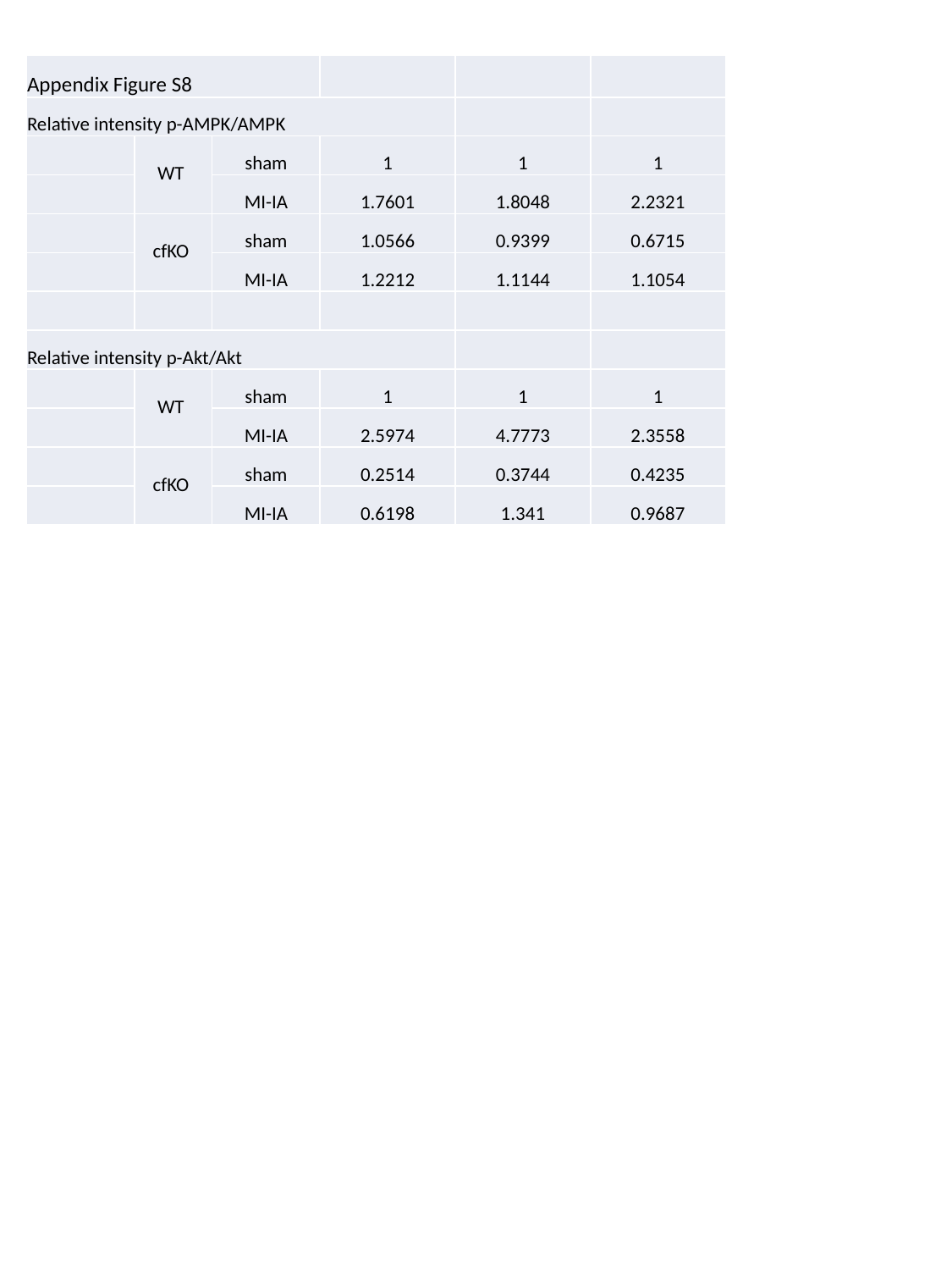

| Appendix Figure S8 | | | | | |
| --- | --- | --- | --- | --- | --- |
| Relative intensity p-AMPK/AMPK | | | | | |
| | WT | sham | 1 | 1 | 1 |
| | | MI-IA | 1.7601 | 1.8048 | 2.2321 |
| | cfKO | sham | 1.0566 | 0.9399 | 0.6715 |
| | | MI-IA | 1.2212 | 1.1144 | 1.1054 |
| | | | | | |
| Relative intensity p-Akt/Akt | | | | | |
| | WT | sham | 1 | 1 | 1 |
| | | MI-IA | 2.5974 | 4.7773 | 2.3558 |
| | cfKO | sham | 0.2514 | 0.3744 | 0.4235 |
| | | MI-IA | 0.6198 | 1.341 | 0.9687 |
