# Supplementary material for: Follistatin‐like 1 promotes cardiac fibroblast activation and protects the heart from rupture
Source: EMBO Mol Med. 2016 May 27;8(8):949–66. doi: 10.15252/emmm.201506151 (PMC4967946; doi:10.15252/emmm.201506151)
Supplement: Supplementary file 8 — Source Data for Expanded View and Appendix [file EMMM-8-949-s008.zip › Source_Data_For_EV_And_Appendix/Figure_EV5_Source_data.pptx]

## Slide 1
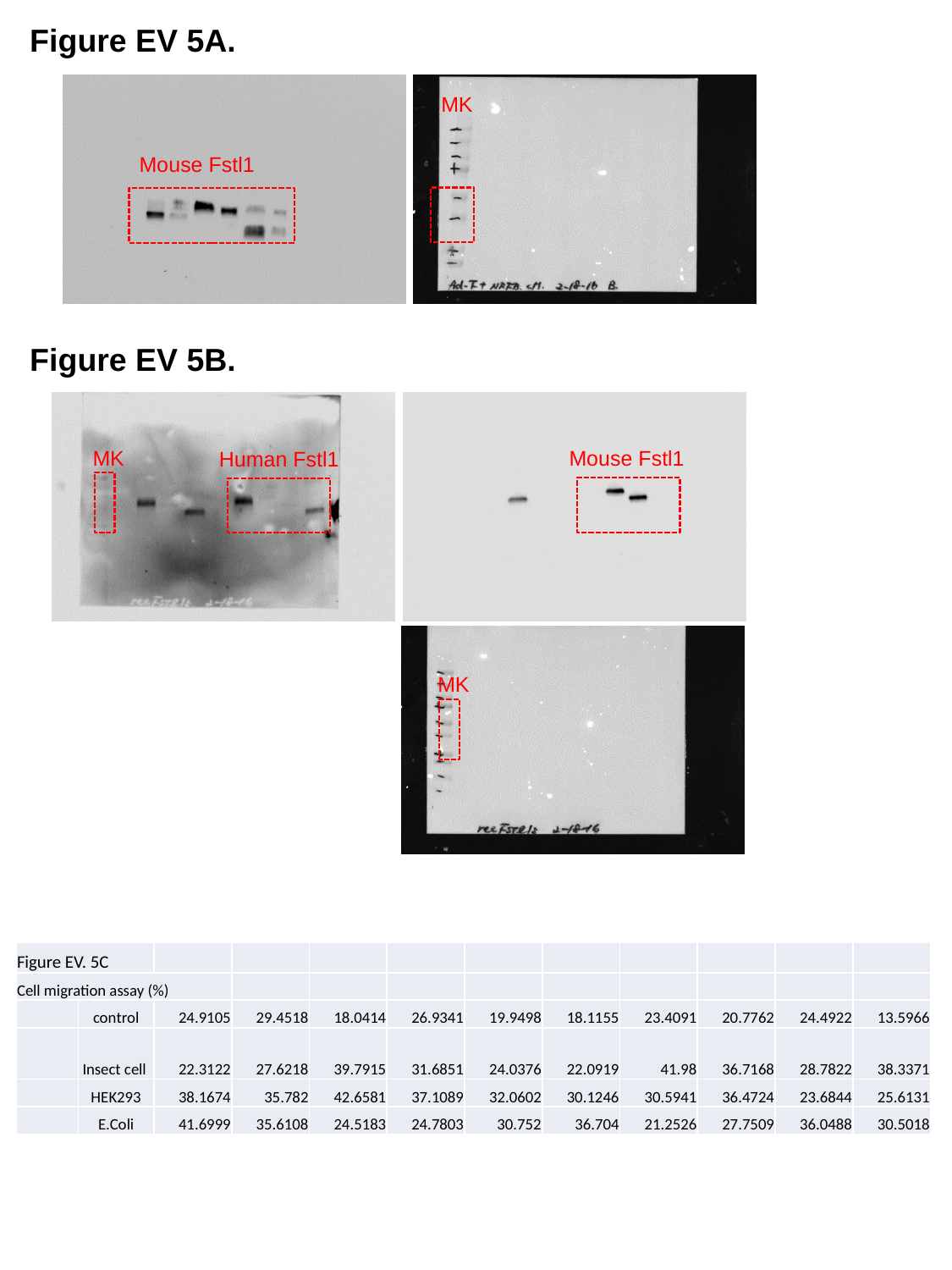

Figure EV 5A.
MK
Mouse Fstl1
Figure EV 5B.
MK
Mouse Fstl1
Human Fstl1
MK
| Figure EV. 5C | | | | | | | | | | | |
| --- | --- | --- | --- | --- | --- | --- | --- | --- | --- | --- | --- |
| Cell migration assay (%) | | | | | | | | | | | |
| | control | 24.9105 | 29.4518 | 18.0414 | 26.9341 | 19.9498 | 18.1155 | 23.4091 | 20.7762 | 24.4922 | 13.5966 |
| | Insect cell | 22.3122 | 27.6218 | 39.7915 | 31.6851 | 24.0376 | 22.0919 | 41.98 | 36.7168 | 28.7822 | 38.3371 |
| | HEK293 | 38.1674 | 35.782 | 42.6581 | 37.1089 | 32.0602 | 30.1246 | 30.5941 | 36.4724 | 23.6844 | 25.6131 |
| | E.Coli | 41.6999 | 35.6108 | 24.5183 | 24.7803 | 30.752 | 36.704 | 21.2526 | 27.7509 | 36.0488 | 30.5018 |
