# Supplementary material for: Follistatin‐like 1 promotes cardiac fibroblast activation and protects the heart from rupture
Source: EMBO Mol Med. 2016 May 27;8(8):949–66. doi: 10.15252/emmm.201506151 (PMC4967946; doi:10.15252/emmm.201506151)
Supplement: Supplementary file 8 — Source Data for Expanded View and Appendix [file EMMM-8-949-s008.zip › Source_Data_For_EV_And_Appendix/Figure_EV3_Source_data.pptx]

## Slide 1
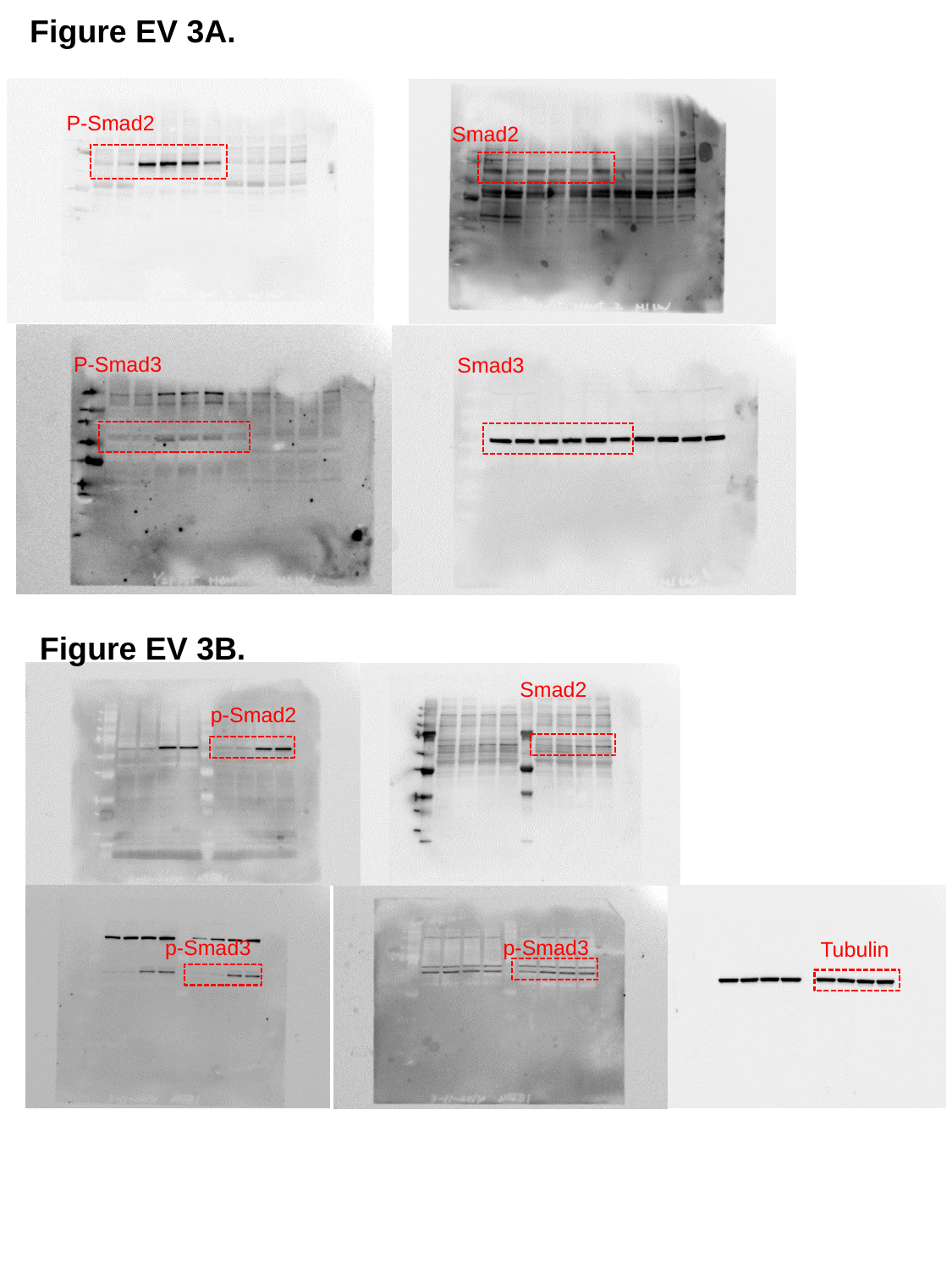

Figure EV 3A.
P-Smad2
Smad2
P-Smad3
Smad3
Figure EV 3B.
p-Smad2
Smad2
Tubulin
p-Smad3
p-Smad3

## Slide 2
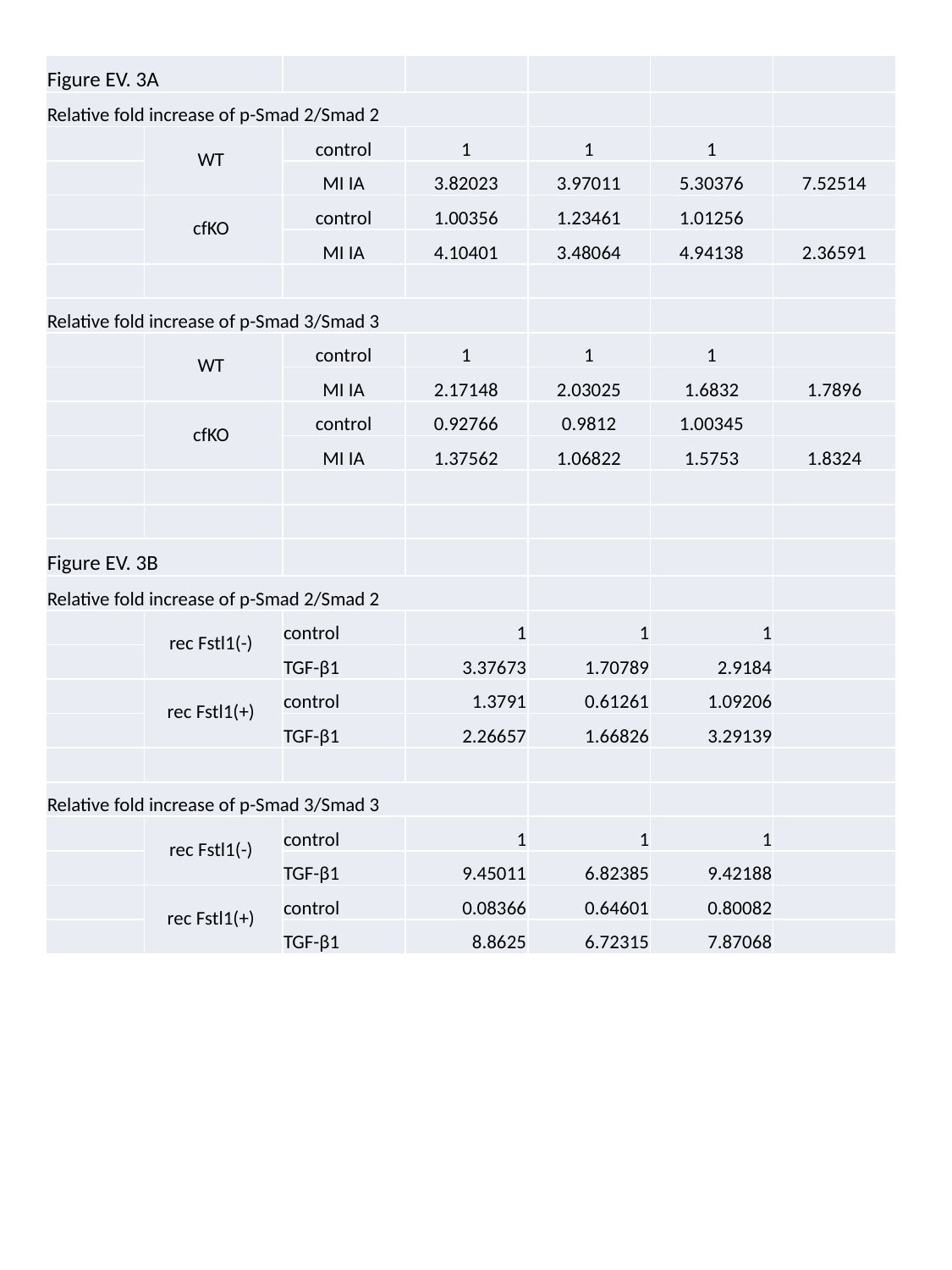

| Figure EV. 3A | | | | | | |
| --- | --- | --- | --- | --- | --- | --- |
| Relative fold increase of p-Smad 2/Smad 2 | | | | | | |
| | WT | control | 1 | 1 | 1 | |
| | | MI IA | 3.82023 | 3.97011 | 5.30376 | 7.52514 |
| | cfKO | control | 1.00356 | 1.23461 | 1.01256 | |
| | | MI IA | 4.10401 | 3.48064 | 4.94138 | 2.36591 |
| | | | | | | |
| Relative fold increase of p-Smad 3/Smad 3 | | | | | | |
| | WT | control | 1 | 1 | 1 | |
| | | MI IA | 2.17148 | 2.03025 | 1.6832 | 1.7896 |
| | cfKO | control | 0.92766 | 0.9812 | 1.00345 | |
| | | MI IA | 1.37562 | 1.06822 | 1.5753 | 1.8324 |
| | | | | | | |
| | | | | | | |
| Figure EV. 3B | | | | | | |
| Relative fold increase of p-Smad 2/Smad 2 | | | | | | |
| | rec Fstl1(-) | control | 1 | 1 | 1 | |
| | | TGF-β1 | 3.37673 | 1.70789 | 2.9184 | |
| | rec Fstl1(+) | control | 1.3791 | 0.61261 | 1.09206 | |
| | | TGF-β1 | 2.26657 | 1.66826 | 3.29139 | |
| | | | | | | |
| Relative fold increase of p-Smad 3/Smad 3 | | | | | | |
| | rec Fstl1(-) | control | 1 | 1 | 1 | |
| | | TGF-β1 | 9.45011 | 6.82385 | 9.42188 | |
| | rec Fstl1(+) | control | 0.08366 | 0.64601 | 0.80082 | |
| | | TGF-β1 | 8.8625 | 6.72315 | 7.87068 | |
