# Supplementary material for: Follistatin‐like 1 promotes cardiac fibroblast activation and protects the heart from rupture
Source: EMBO Mol Med. 2016 May 27;8(8):949–66. doi: 10.15252/emmm.201506151 (PMC4967946; doi:10.15252/emmm.201506151)
Supplement: Supplementary file 8 — Source Data for Expanded View and Appendix [file EMMM-8-949-s008.zip › Source_Data_For_EV_And_Appendix/Figure_EV4_Source_data.pptx]

## Slide 1
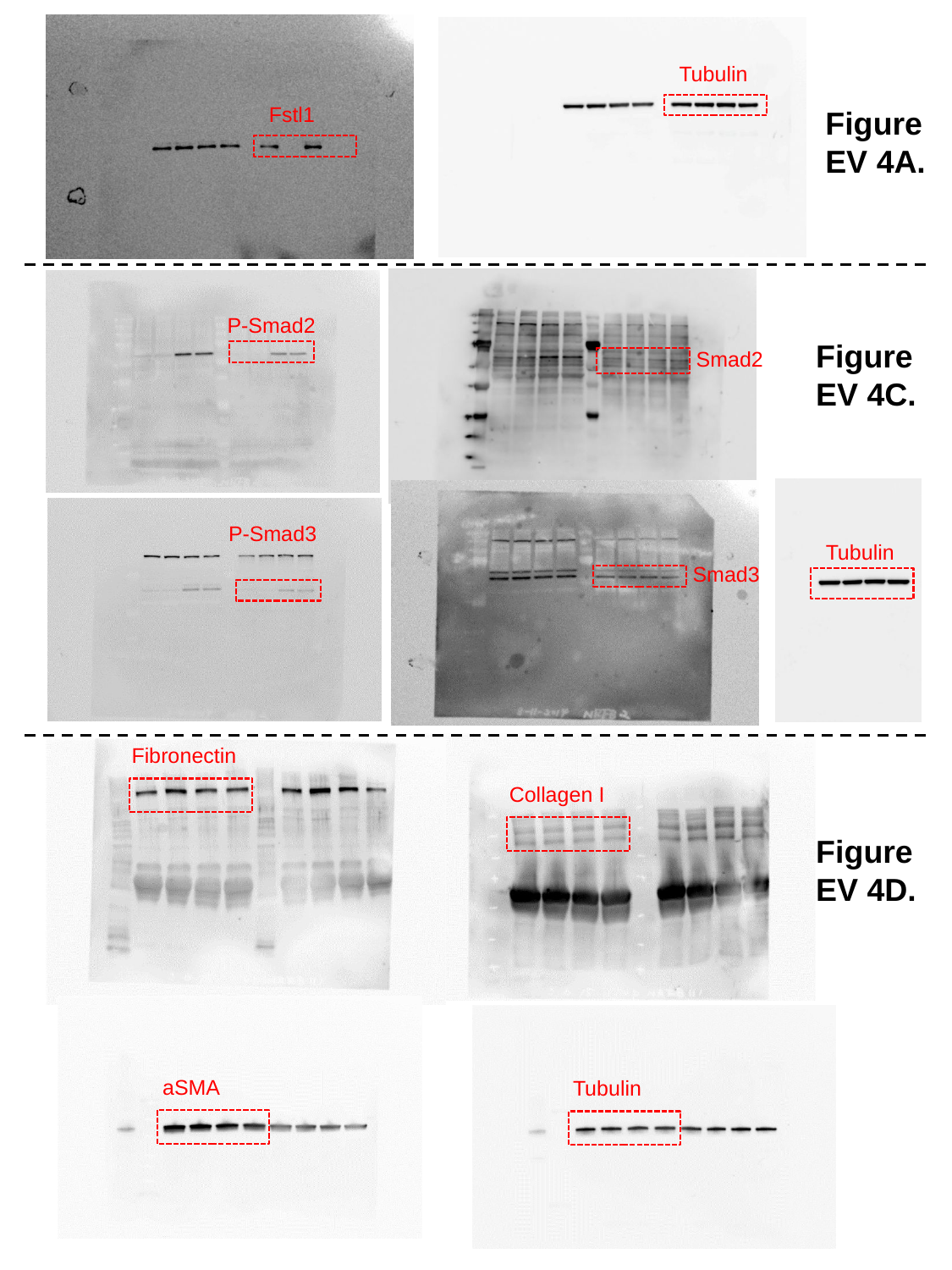

Fstl1
Tubulin
Figure
EV 4A.
Smad2
P-Smad2
Figure EV 4C.
Tubulin
Smad3
P-Smad3
Collagen I
Fibronectin
Figure EV 4D.
aSMA
Tubulin

## Slide 2
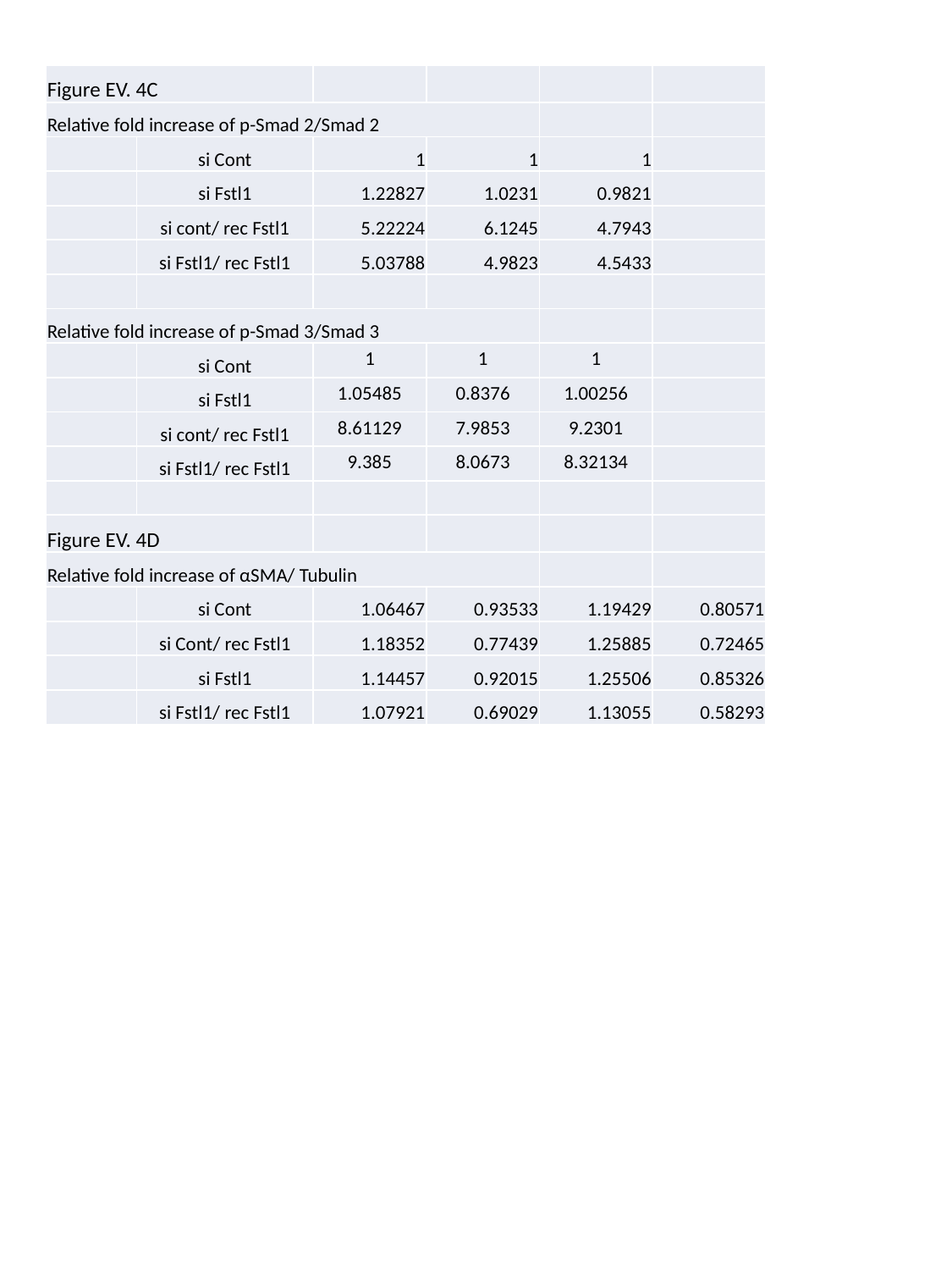

| Figure EV. 4C | | | | | |
| --- | --- | --- | --- | --- | --- |
| Relative fold increase of p-Smad 2/Smad 2 | | | | | |
| | si Cont | 1 | 1 | 1 | |
| | si Fstl1 | 1.22827 | 1.0231 | 0.9821 | |
| | si cont/ rec Fstl1 | 5.22224 | 6.1245 | 4.7943 | |
| | si Fstl1/ rec Fstl1 | 5.03788 | 4.9823 | 4.5433 | |
| | | | | | |
| Relative fold increase of p-Smad 3/Smad 3 | | | | | |
| | si Cont | 1 | 1 | 1 | |
| | si Fstl1 | 1.05485 | 0.8376 | 1.00256 | |
| | si cont/ rec Fstl1 | 8.61129 | 7.9853 | 9.2301 | |
| | si Fstl1/ rec Fstl1 | 9.385 | 8.0673 | 8.32134 | |
| | | | | | |
| Figure EV. 4D | | | | | |
| Relative fold increase of αSMA/ Tubulin | | | | | |
| | si Cont | 1.06467 | 0.93533 | 1.19429 | 0.80571 |
| | si Cont/ rec Fstl1 | 1.18352 | 0.77439 | 1.25885 | 0.72465 |
| | si Fstl1 | 1.14457 | 0.92015 | 1.25506 | 0.85326 |
| | si Fstl1/ rec Fstl1 | 1.07921 | 0.69029 | 1.13055 | 0.58293 |
