# Supplementary figures and images for: Follistatin‐like 1 promotes cardiac fibroblast activation and protects the heart from rupture
Source: EMBO Mol Med. 2016 May 27;8(8):949–66. doi: 10.15252/emmm.201506151 (PMC4967946; doi:10.15252/emmm.201506151)

## Slide 1
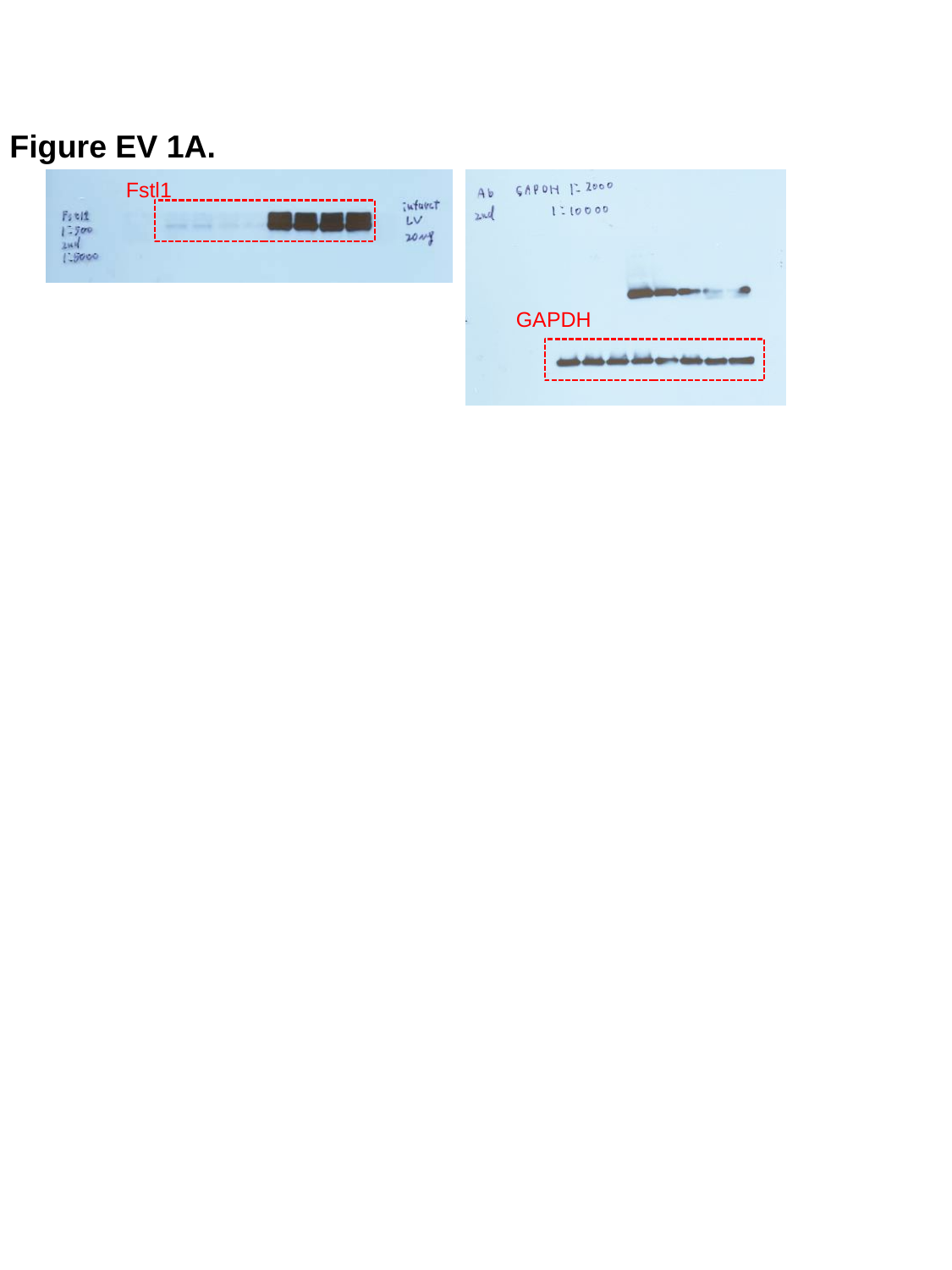

Figure EV 1A.
Fstl1
GAPDH

Supplement: Supplementary file 8 — Source Data for Expanded View and Appendix [file EMMM-8-949-s008.zip › Source_Data_For_EV_And_Appendix/Figure_EV1_Source_data.pptx]
