# Supplementary material for: Follistatin‐like 1 promotes cardiac fibroblast activation and protects the heart from rupture
Source: EMBO Mol Med. 2016 May 27;8(8):949–66. doi: 10.15252/emmm.201506151 (PMC4967946; doi:10.15252/emmm.201506151)
Supplement: Supplementary file 10 — Source Data for Figure 1 [file EMMM-8-949-s009.pptx]

## Slide 1
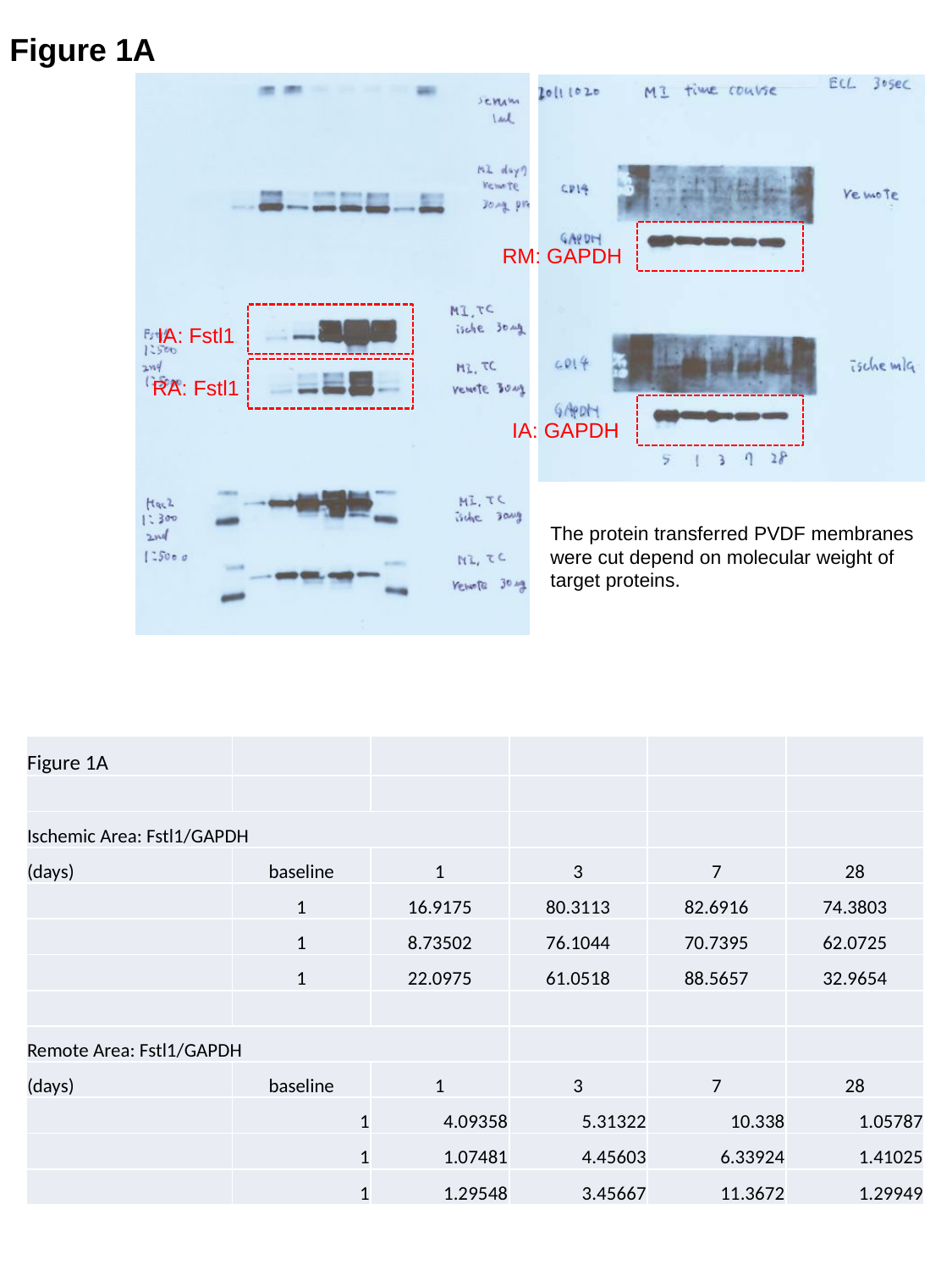

Figure 1A
IA: Fstl1
RA: Fstl1
RM: GAPDH
IA: GAPDH
The protein transferred PVDF membranes were cut depend on molecular weight of target proteins.
| Figure 1A | | | | | |
| --- | --- | --- | --- | --- | --- |
| | | | | | |
| Ischemic Area: Fstl1/GAPDH | | | | | |
| (days) | baseline | 1 | 3 | 7 | 28 |
| | 1 | 16.9175 | 80.3113 | 82.6916 | 74.3803 |
| | 1 | 8.73502 | 76.1044 | 70.7395 | 62.0725 |
| | 1 | 22.0975 | 61.0518 | 88.5657 | 32.9654 |
| | | | | | |
| Remote Area: Fstl1/GAPDH | | | | | |
| (days) | baseline | 1 | 3 | 7 | 28 |
| | 1 | 4.09358 | 5.31322 | 10.338 | 1.05787 |
| | 1 | 1.07481 | 4.45603 | 6.33924 | 1.41025 |
| | 1 | 1.29548 | 3.45667 | 11.3672 | 1.29949 |

## Slide 2
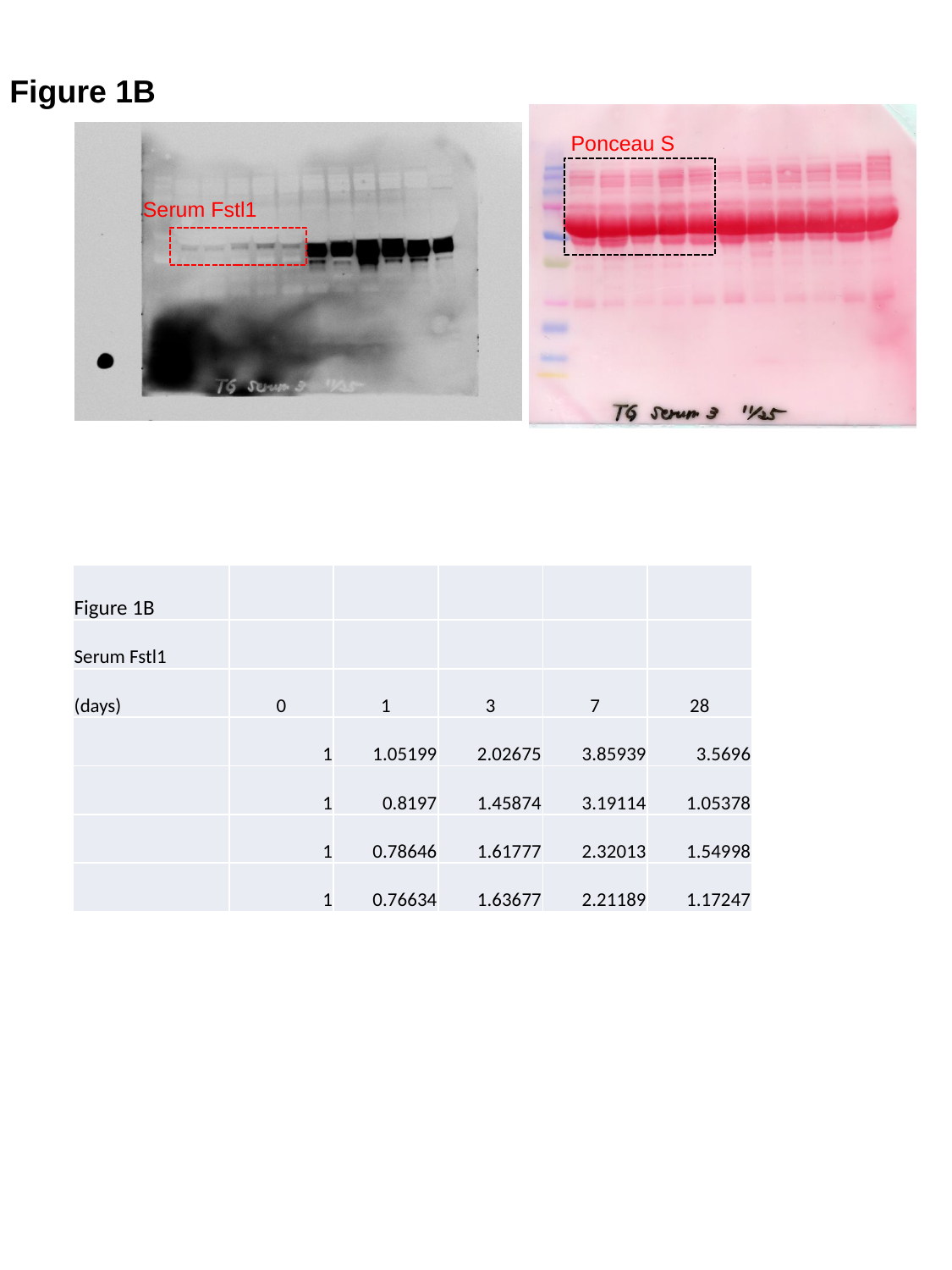

Figure 1B
Ponceau S
Serum Fstl1
| Figure 1B | | | | | |
| --- | --- | --- | --- | --- | --- |
| Serum Fstl1 | | | | | |
| (days) | 0 | 1 | 3 | 7 | 28 |
| | 1 | 1.05199 | 2.02675 | 3.85939 | 3.5696 |
| | 1 | 0.8197 | 1.45874 | 3.19114 | 1.05378 |
| | 1 | 0.78646 | 1.61777 | 2.32013 | 1.54998 |
| | 1 | 0.76634 | 1.63677 | 2.21189 | 1.17247 |
