# Supplementary material for: Follistatin‐like 1 promotes cardiac fibroblast activation and protects the heart from rupture
Source: EMBO Mol Med. 2016 May 27;8(8):949–66. doi: 10.15252/emmm.201506151 (PMC4967946; doi:10.15252/emmm.201506151)
Supplement: Supplementary file 13 — Source Data for Figure 5 [file EMMM-8-949-s012.pptx]

## Slide 1
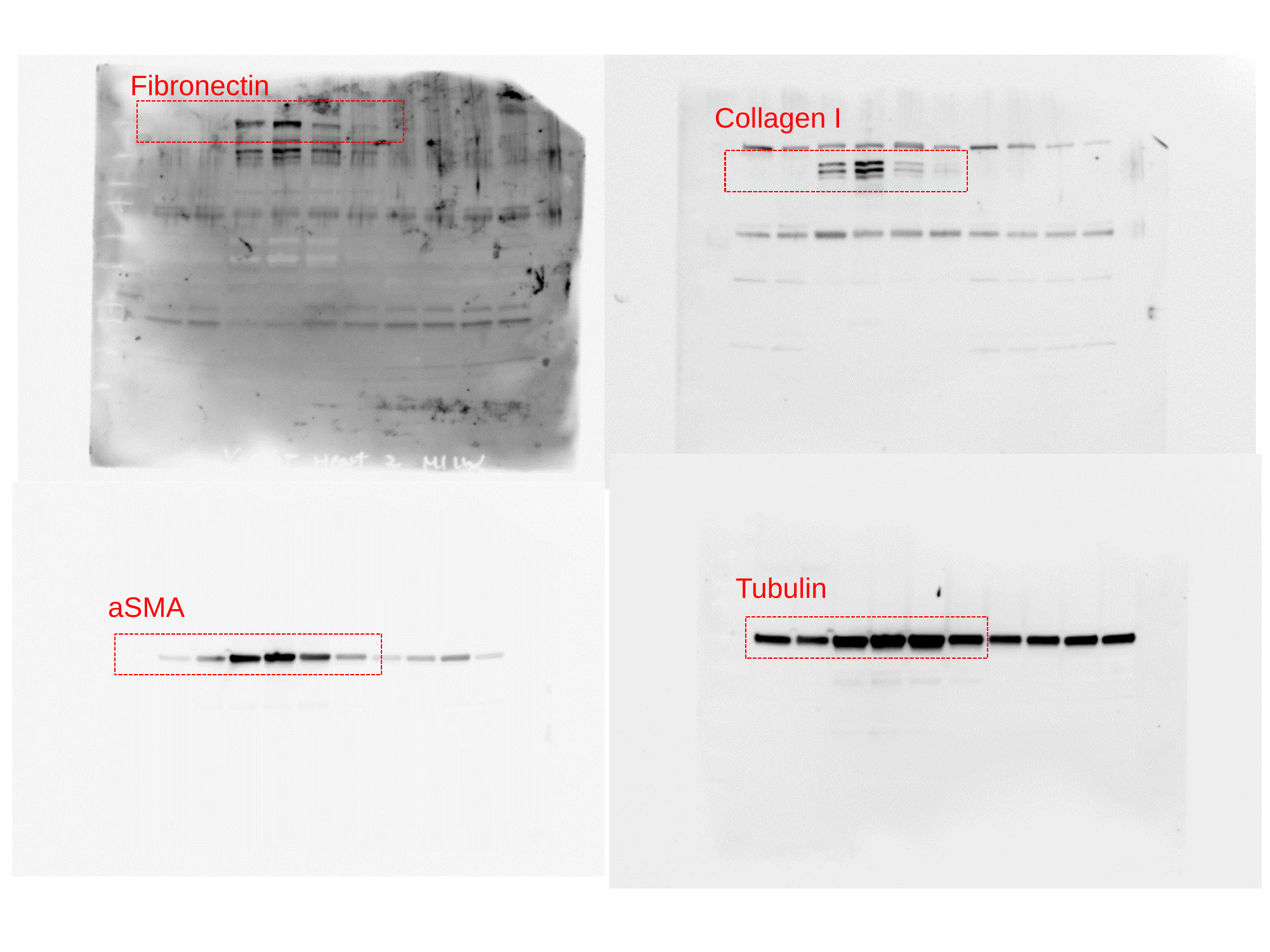

Figure 5B
Fibronectin
Collagen I
Tubulin
aSMA

## Slide 2
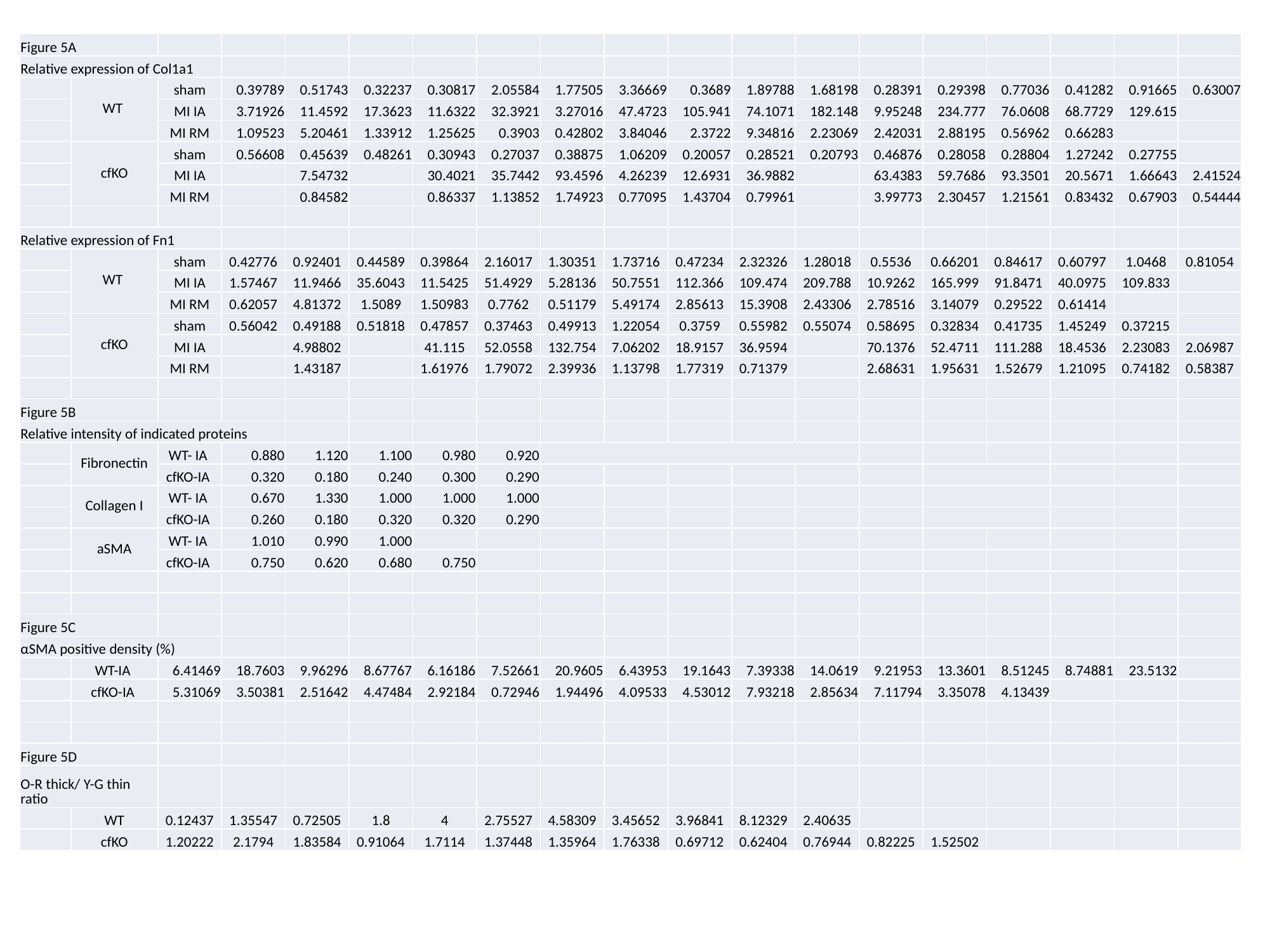

| Figure 5A | | | | | | | | | | | | | | | | | | |
| --- | --- | --- | --- | --- | --- | --- | --- | --- | --- | --- | --- | --- | --- | --- | --- | --- | --- | --- |
| Relative expression of Col1a1 | | | | | | | | | | | | | | | | | | |
| | WT | sham | 0.39789 | 0.51743 | 0.32237 | 0.30817 | 2.05584 | 1.77505 | 3.36669 | 0.3689 | 1.89788 | 1.68198 | 0.28391 | 0.29398 | 0.77036 | 0.41282 | 0.91665 | 0.63007 |
| | | MI IA | 3.71926 | 11.4592 | 17.3623 | 11.6322 | 32.3921 | 3.27016 | 47.4723 | 105.941 | 74.1071 | 182.148 | 9.95248 | 234.777 | 76.0608 | 68.7729 | 129.615 | |
| | | MI RM | 1.09523 | 5.20461 | 1.33912 | 1.25625 | 0.3903 | 0.42802 | 3.84046 | 2.3722 | 9.34816 | 2.23069 | 2.42031 | 2.88195 | 0.56962 | 0.66283 | | |
| | cfKO | sham | 0.56608 | 0.45639 | 0.48261 | 0.30943 | 0.27037 | 0.38875 | 1.06209 | 0.20057 | 0.28521 | 0.20793 | 0.46876 | 0.28058 | 0.28804 | 1.27242 | 0.27755 | |
| | | MI IA | | 7.54732 | | 30.4021 | 35.7442 | 93.4596 | 4.26239 | 12.6931 | 36.9882 | | 63.4383 | 59.7686 | 93.3501 | 20.5671 | 1.66643 | 2.41524 |
| | | MI RM | | 0.84582 | | 0.86337 | 1.13852 | 1.74923 | 0.77095 | 1.43704 | 0.79961 | | 3.99773 | 2.30457 | 1.21561 | 0.83432 | 0.67903 | 0.54444 |
| | | | | | | | | | | | | | | | | | | |
| Relative expression of Fn1 | | | | | | | | | | | | | | | | | | |
| | WT | sham | 0.42776 | 0.92401 | 0.44589 | 0.39864 | 2.16017 | 1.30351 | 1.73716 | 0.47234 | 2.32326 | 1.28018 | 0.5536 | 0.66201 | 0.84617 | 0.60797 | 1.0468 | 0.81054 |
| | | MI IA | 1.57467 | 11.9466 | 35.6043 | 11.5425 | 51.4929 | 5.28136 | 50.7551 | 112.366 | 109.474 | 209.788 | 10.9262 | 165.999 | 91.8471 | 40.0975 | 109.833 | |
| | | MI RM | 0.62057 | 4.81372 | 1.5089 | 1.50983 | 0.7762 | 0.51179 | 5.49174 | 2.85613 | 15.3908 | 2.43306 | 2.78516 | 3.14079 | 0.29522 | 0.61414 | | |
| | cfKO | sham | 0.56042 | 0.49188 | 0.51818 | 0.47857 | 0.37463 | 0.49913 | 1.22054 | 0.3759 | 0.55982 | 0.55074 | 0.58695 | 0.32834 | 0.41735 | 1.45249 | 0.37215 | |
| | | MI IA | | 4.98802 | | 41.115 | 52.0558 | 132.754 | 7.06202 | 18.9157 | 36.9594 | | 70.1376 | 52.4711 | 111.288 | 18.4536 | 2.23083 | 2.06987 |
| | | MI RM | | 1.43187 | | 1.61976 | 1.79072 | 2.39936 | 1.13798 | 1.77319 | 0.71379 | | 2.68631 | 1.95631 | 1.52679 | 1.21095 | 0.74182 | 0.58387 |
| | | | | | | | | | | | | | | | | | | |
| Figure 5B | | | | | | | | | | | | | | | | | | |
| Relative intensity of indicated proteins | | | | | | | | | | | | | | | | | | |
| | Fibronectin | WT- IA | 0.880 | 1.120 | 1.100 | 0.980 | 0.920 | | | | | | | | | | | |
| | | cfKO-IA | 0.320 | 0.180 | 0.240 | 0.300 | 0.290 | | | | | | | | | | | |
| | Collagen I | WT- IA | 0.670 | 1.330 | 1.000 | 1.000 | 1.000 | | | | | | | | | | | |
| | | cfKO-IA | 0.260 | 0.180 | 0.320 | 0.320 | 0.290 | | | | | | | | | | | |
| | aSMA | WT- IA | 1.010 | 0.990 | 1.000 | | | | | | | | | | | | | |
| | | cfKO-IA | 0.750 | 0.620 | 0.680 | 0.750 | | | | | | | | | | | | |
| | | | | | | | | | | | | | | | | | | |
| | | | | | | | | | | | | | | | | | | |
| Figure 5C | | | | | | | | | | | | | | | | | | |
| αSMA positive density (%) | | | | | | | | | | | | | | | | | | |
| | WT-IA | 6.41469 | 18.7603 | 9.96296 | 8.67767 | 6.16186 | 7.52661 | 20.9605 | 6.43953 | 19.1643 | 7.39338 | 14.0619 | 9.21953 | 13.3601 | 8.51245 | 8.74881 | 23.5132 | |
| | cfKO-IA | 5.31069 | 3.50381 | 2.51642 | 4.47484 | 2.92184 | 0.72946 | 1.94496 | 4.09533 | 4.53012 | 7.93218 | 2.85634 | 7.11794 | 3.35078 | 4.13439 | | | |
| | | | | | | | | | | | | | | | | | | |
| | | | | | | | | | | | | | | | | | | |
| Figure 5D | | | | | | | | | | | | | | | | | | |
| O-R thick/ Y-G thin ratio | | | | | | | | | | | | | | | | | | |
| | WT | 0.12437 | 1.35547 | 0.72505 | 1.8 | 4 | 2.75527 | 4.58309 | 3.45652 | 3.96841 | 8.12329 | 2.40635 | | | | | | |
| | cfKO | 1.20222 | 2.1794 | 1.83584 | 0.91064 | 1.7114 | 1.37448 | 1.35964 | 1.76338 | 0.69712 | 0.62404 | 0.76944 | 0.82225 | 1.52502 | | | | |
