# Supplementary material for: Follistatin‐like 1 promotes cardiac fibroblast activation and protects the heart from rupture
Source: EMBO Mol Med. 2016 May 27;8(8):949–66. doi: 10.15252/emmm.201506151 (PMC4967946; doi:10.15252/emmm.201506151)
Supplement: Supplementary file 14 — Source Data for Figure 6 [file EMMM-8-949-s013.pptx]

## Slide 1
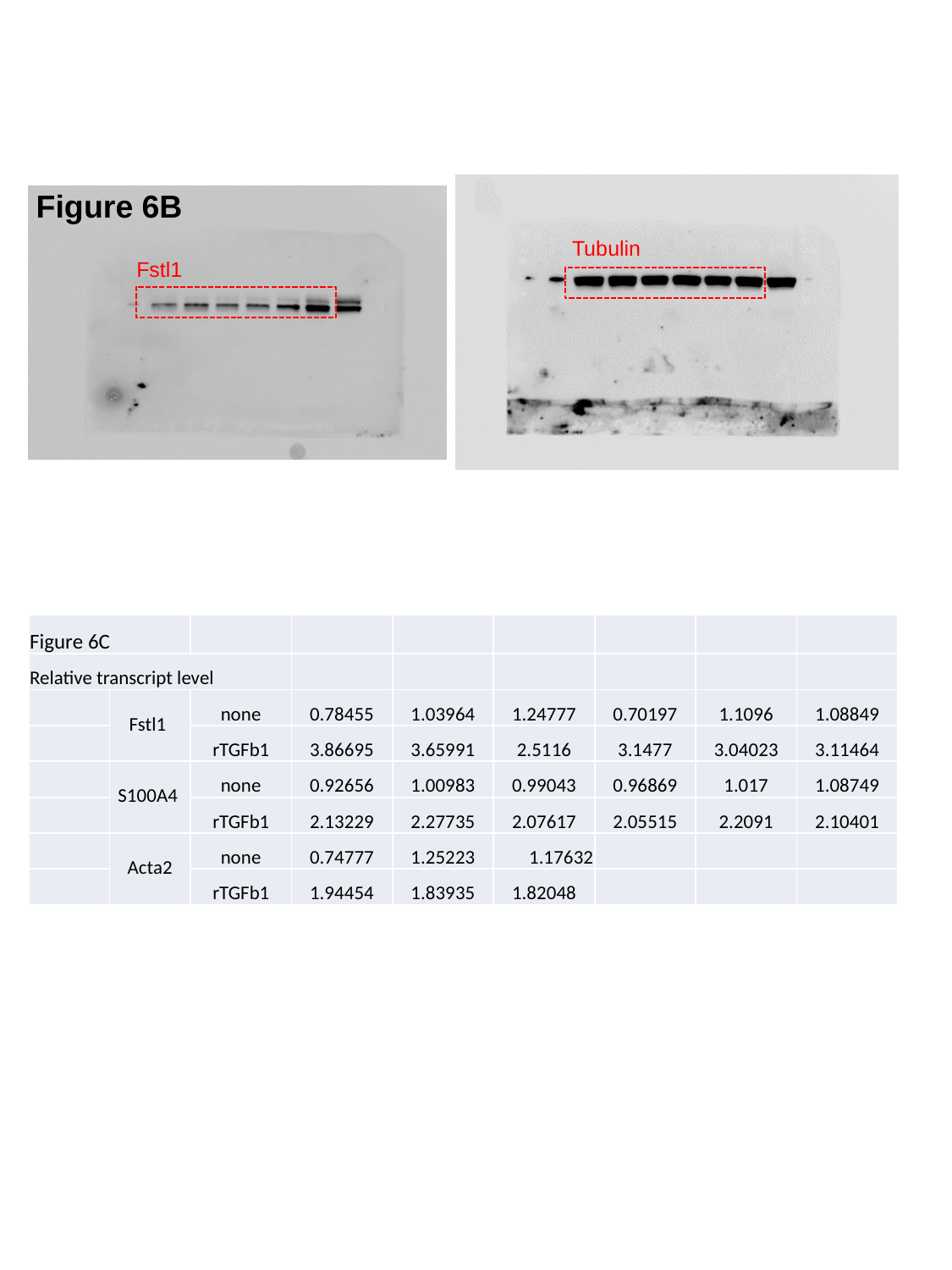

Tubulin
Figure 6B
Fstl1
| Figure 6C | | | | | | | | |
| --- | --- | --- | --- | --- | --- | --- | --- | --- |
| Relative transcript level | | | | | | | | |
| | Fstl1 | none | 0.78455 | 1.03964 | 1.24777 | 0.70197 | 1.1096 | 1.08849 |
| | | rTGFb1 | 3.86695 | 3.65991 | 2.5116 | 3.1477 | 3.04023 | 3.11464 |
| | S100A4 | none | 0.92656 | 1.00983 | 0.99043 | 0.96869 | 1.017 | 1.08749 |
| | | rTGFb1 | 2.13229 | 2.27735 | 2.07617 | 2.05515 | 2.2091 | 2.10401 |
| | Acta2 | none | 0.74777 | 1.25223 | 1.17632 | | | |
| | | rTGFb1 | 1.94454 | 1.83935 | 1.82048 | | | |
