# Supplementary material for: Follistatin‐like 1 promotes cardiac fibroblast activation and protects the heart from rupture
Source: EMBO Mol Med. 2016 May 27;8(8):949–66. doi: 10.15252/emmm.201506151 (PMC4967946; doi:10.15252/emmm.201506151)
Supplement: Supplementary file 15 — Source Data for Figure 7 [file EMMM-8-949-s014.pptx]

## Slide 1
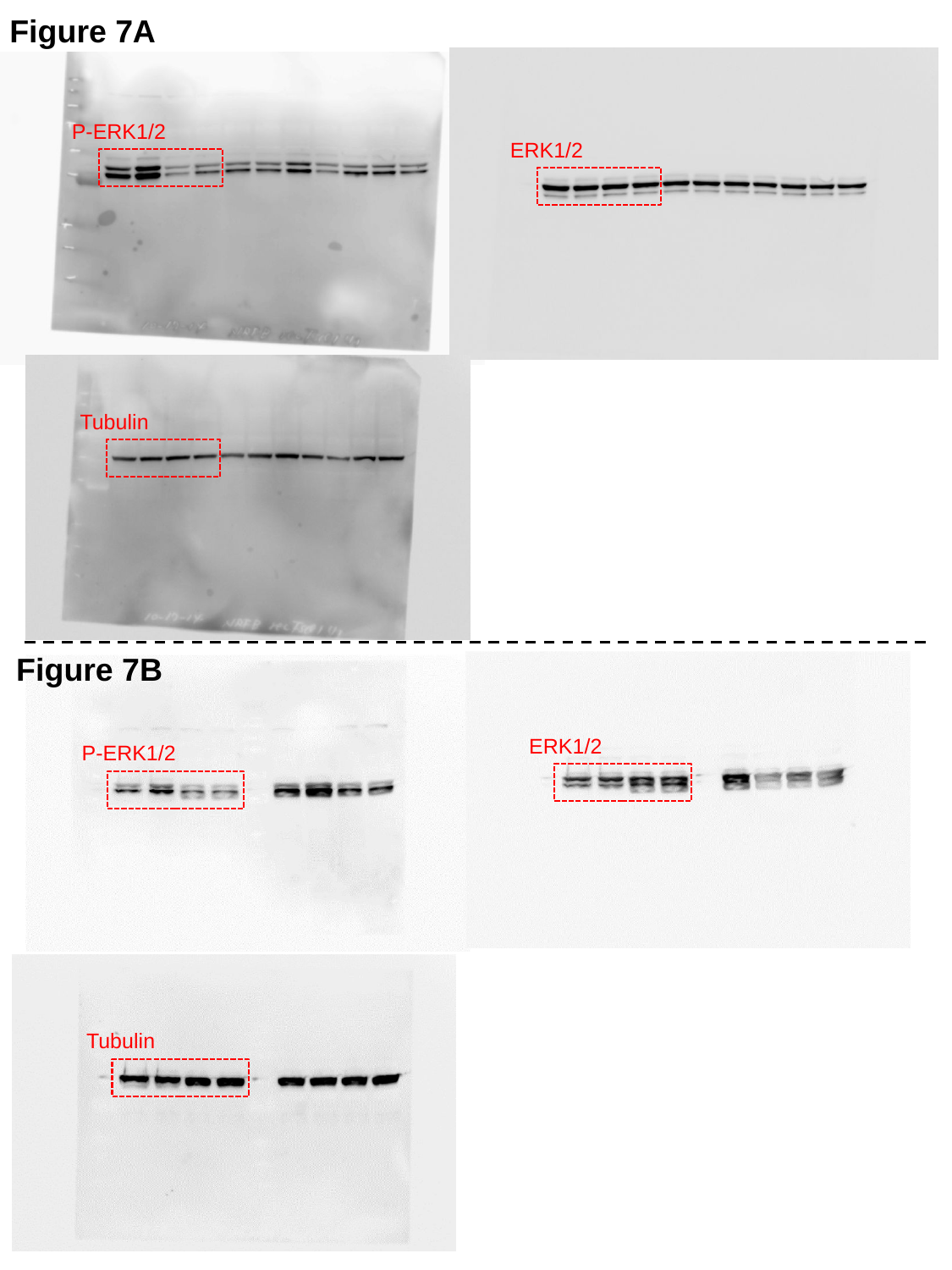

Figure 7A
ERK1/2
P-ERK1/2
Tubulin
Figure 7B
ERK1/2
P-ERK1/2
Tubulin

## Slide 2
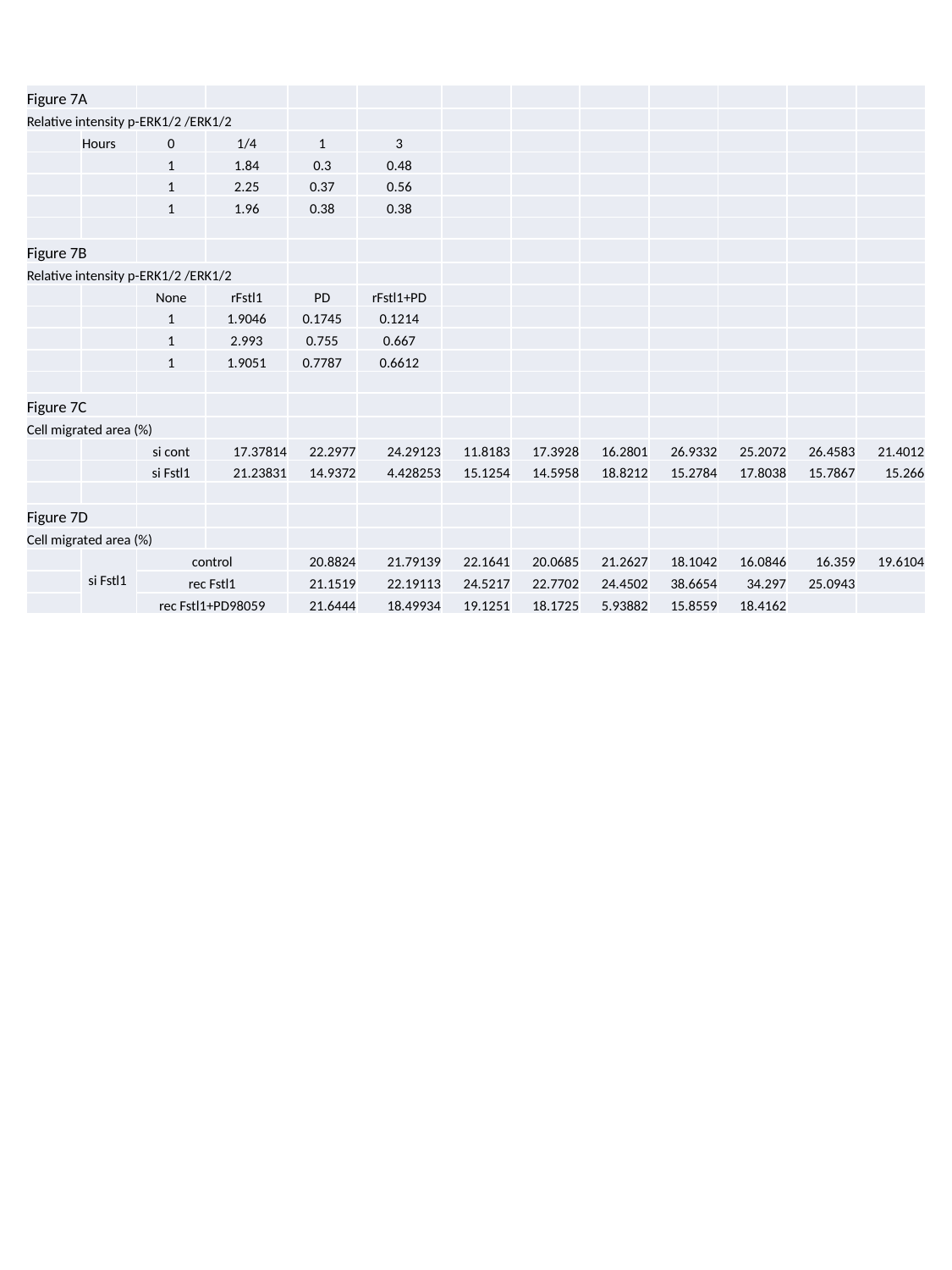

| Figure 7A | | | | | | | | | | | | |
| --- | --- | --- | --- | --- | --- | --- | --- | --- | --- | --- | --- | --- |
| Relative intensity p-ERK1/2 /ERK1/2 | | | | | | | | | | | | |
| | Hours | 0 | 1/4 | 1 | 3 | | | | | | | |
| | | 1 | 1.84 | 0.3 | 0.48 | | | | | | | |
| | | 1 | 2.25 | 0.37 | 0.56 | | | | | | | |
| | | 1 | 1.96 | 0.38 | 0.38 | | | | | | | |
| | | | | | | | | | | | | |
| Figure 7B | | | | | | | | | | | | |
| Relative intensity p-ERK1/2 /ERK1/2 | | | | | | | | | | | | |
| | | None | rFstl1 | PD | rFstl1+PD | | | | | | | |
| | | 1 | 1.9046 | 0.1745 | 0.1214 | | | | | | | |
| | | 1 | 2.993 | 0.755 | 0.667 | | | | | | | |
| | | 1 | 1.9051 | 0.7787 | 0.6612 | | | | | | | |
| | | | | | | | | | | | | |
| Figure 7C | | | | | | | | | | | | |
| Cell migrated area (%) | | | | | | | | | | | | |
| | | si cont | 17.37814 | 22.2977 | 24.29123 | 11.8183 | 17.3928 | 16.2801 | 26.9332 | 25.2072 | 26.4583 | 21.4012 |
| | | si Fstl1 | 21.23831 | 14.9372 | 4.428253 | 15.1254 | 14.5958 | 18.8212 | 15.2784 | 17.8038 | 15.7867 | 15.266 |
| | | | | | | | | | | | | |
| Figure 7D | | | | | | | | | | | | |
| Cell migrated area (%) | | | | | | | | | | | | |
| | si Fstl1 | control | | 20.8824 | 21.79139 | 22.1641 | 20.0685 | 21.2627 | 18.1042 | 16.0846 | 16.359 | 19.6104 |
| | | rec Fstl1 | | 21.1519 | 22.19113 | 24.5217 | 22.7702 | 24.4502 | 38.6654 | 34.297 | 25.0943 | |
| | | rec Fstl1+PD98059 | | 21.6444 | 18.49934 | 19.1251 | 18.1725 | 5.93882 | 15.8559 | 18.4162 | | |
